# The effectiveness of computerized order entry at reducing preventable adverse drug events and medication errors in hospital settings: a systematic review and meta-analysis

**DOI:** 10.1186/2046-4053-3-56

**Published:** 2014-06-04

**Authors:** Teryl K Nuckols, Crystal Smith-Spangler, Sally C Morton, Steven M Asch, Vaspaan M Patel, Laura J Anderson, Emily L Deichsel, Paul G Shekelle

**Affiliations:** 1 Division of General Internal Medicine and Health Services Research, David Geffen School of Medicine at the University of California, 911 Broxton Ave, Los Angeles, CA 90024, USA; 2 RAND Corporation, 1776 Main Street, Santa Monica, CA 90407, USA; 3 VA Palo Alto Health Care System, 795 Willow Road, Menlo Park, CA 94025, USA; 4 Stanford University, Palo Alto, CA 94305, USA; 5 Department of Biostatistics, University of Pittsburgh, Graduate School of Public Health, Pittsburgh, PA 15261, USA; 6 NCQA, 1100 13th street NW, Washington, DC 20005, USA; 7 UCLA Jonathan and Karin Fielding School of Public Health, Los Angeles, CA 90024, USA; 8 VA Greater Los Angeles Healthcare System, Los Angeles, CA, USA

**Keywords:** Medical order entry systems, Drug toxicity/prevention and control, Hospitals, Adverse drug event, Medication error

## Abstract

**Background:**

The Health Information Technology for Economic and Clinical Health (HITECH) Act subsidizes implementation by hospitals of electronic health records with computerized provider order entry (CPOE), which may reduce patient injuries caused by medication errors (preventable adverse drug events, pADEs). Effects on pADEs have not been rigorously quantified, and effects on medication errors have been variable. The objectives of this analysis were to assess the effectiveness of CPOE at reducing pADEs in hospital-related settings, and examine reasons for heterogeneous effects on medication errors.

**Methods:**

Articles were identified using MEDLINE, Cochrane Library, Econlit, web-based databases, and bibliographies of previous systematic reviews (September 2013). Eligible studies compared CPOE with paper-order entry in acute care hospitals, and examined diverse pADEs or medication errors. Studies on children or with limited event-detection methods were excluded. Two investigators extracted data on events and factors potentially associated with effectiveness. We used random effects models to pool data.

**Results:**

Sixteen studies addressing medication errors met pooling criteria; six also addressed pADEs. Thirteen studies used pre-post designs. Compared with paper-order entry, CPOE was associated with half as many pADEs (pooled risk ratio (RR) = 0.47, 95% CI 0.31 to 0.71) and medication errors (RR = 0.46, 95% CI 0.35 to 0.60). Regarding reasons for heterogeneous effects on medication errors, five intervention factors and two contextual factors were sufficiently reported to support subgroup analyses or meta-regression. Differences between commercial versus homegrown systems, presence and sophistication of clinical decision support, hospital-wide versus limited implementation, and US versus non-US studies were not significant, nor was timing of publication. Higher baseline rates of medication errors predicted greater reductions (*P* < 0.001). Other context and implementation variables were seldom reported.

**Conclusions:**

In hospital-related settings, implementing CPOE is associated with a greater than 50% decline in pADEs, although the studies used weak designs. Decreases in medication errors are similar and robust to variations in important aspects of intervention design and context. This suggests that CPOE implementation, as subsidized under the HITECH Act, may benefit public health. More detailed reporting of the context and process of implementation could shed light on factors associated with greater effectiveness.

## Background

The Health Information Technology for Economic and Clinical Health (HITECH) Act of 2009 incentivizes the adoption of health information technology by US hospitals. This Act, part of the American Reinvestment and Recovery Act, allocates up to $29 billion over 10 years for the implementation and 'meaningful use' of electronic health records by hospitals and healthcare providers [1]. Hospitals that satisfy meaningful use criteria can receive millions of dollars. Implementing computerized provider order entry (CPOE) with clinical decision support systems (CDSS) that check for allergies and drug-drug interactions is one of several basic (Stage 1) criteria for meaningful use by hospitals [2]. As of 2008, approximately 9% of general acute care hospitals had at least basic electronic health record (EHR) systems including CPOE for medications. By 2012, 44% had such systems, specifically, 38% of small, 47% of medium, and 62% of large hospitals [3]. Thus, despite the financial incentives, about half of small and medium hospitals and almost 40% of large hospitals had not adopted CPOE with CDSS in the most recent survey.

The primary potential benefit of adopting CPOE is reducing patient injuries caused by medication errors, called preventable adverse drug events (pADEs) [4-6]. Counterbalancing this is concern about unintended adverse consequences [7-9], including increases in medication errors and even mortality, which have been detected in some hospitals after implementation of CPOE [10,11]. To date, no systematic review has examined net effects on pADEs, the primary outcome of interest for this intervention. Previous reviews have, instead, focused almost exclusively on an intermediate outcome, medication errors. However, not all medication errors pose an equal risk of causing injury. Errors in timing, for example, are generally less risky than giving a medication to the wrong patient. Many commonly used medications, such as anti-hypertensives and antibiotics, have sufficiently long half-lives that receiving a dose an hour or two late has little clinical effect. By contrast, receiving an anti-hypertensive or antibiotic intended for someone else poses risks of low blood pressure or an allergic reaction. In one study at six hospitals, only about 20% of medication errors led to pADEs [12]. Thus, the effect of CPOE on the patient outcome of pADEs is an important clinical and policy question that has remained unanswered, until now.

In addition to focusing on medication errors rather than pADEs, previous systematic reviews have reached conflicting conclusions about the effects of CPOE on medication errors in acute care settings. Some have concluded that CPOE reduces errors, whereas others argue that net effects remain uncertain [4,5,13-42]. This controversy stems, in part, from the fact that the association between CPOE implementation and medication errors has exhibited substantial heterogeneity across primary studies [37]. Three basic types of factors could explain such variability: intervention factors, such as differences in how the intervention is designed and implemented; contextual factors, such as differences in patient populations and settings; and methodological factors, such as differences in study design and execution [43].

Uncertainty about the effects of CPOE on patient outcomes and its variable effects on medication errors may contribute to the reluctance of some hospitals and physicians to adopt CPOE, despite the financial incentives available via HITECH. Consequently, our primary objective in this study was to quantitatively assess the effectiveness of CPOE at reducing pADEs in hospital-related acute care settings. Our secondary objective was to identify factors contributing to variability in effectiveness at reducing medication errors. This analysis is timely as several studies have been published recently and, therefore, were not included in previous reviews and meta-analyses [4,13,34,37,41], enabling us to examine effects on pADEs and reasons for heterogeneity.

## Methods

We adhered to recommendations in the Preferred Reporting Items for Systematic Reviews and Meta-Analyses (PRISMA) Statement [44,45], including developing the protocol before undertaking the analysis.

### Data sources and searches

First, we developed search strategies for eight databases: MEDLINE; Cochrane Library; Econlit; Campbell Collaboration; the Agency for Healthcare Research and Quality (AHRQ) Health Information Technology Library, Health Information Technology Bibliography, Health Information Technology Costs and Benefits Database Project, and PSNET; Information Service Center for Reviews and Dissemination at the University of York; Evidence for Policy and Practice Information and Coordinating Centre (EPPI-Centre), University of London; Oregon Health Sciences Searchable CPOE Bibliography; and Health Systems Evidence, McMaster University. A number of search terms, such as 'order entry' and 'electronic prescribing' (see Additional file 1), were chosen and strategies developed, in part based on a search strategy published by Eslami *et al*. [4].

**Figure 1:**
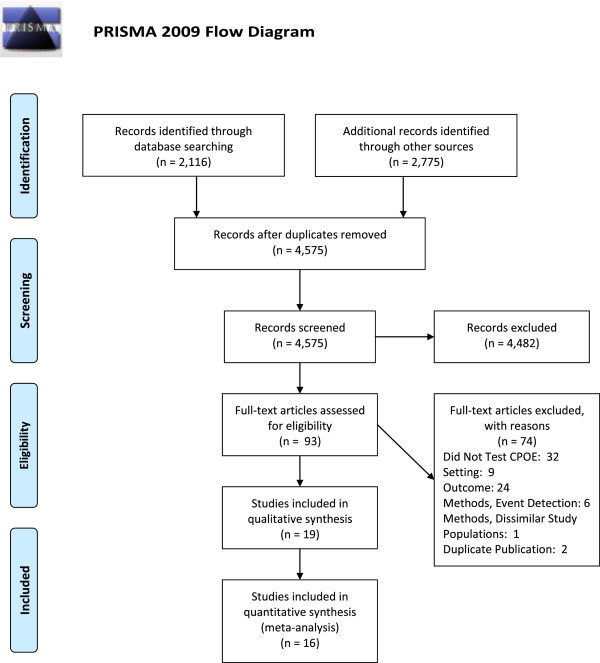
Summary of evidence search and selection.

We used this strategy to search the eight databases for systematic reviews of CPOE or CDSS that might contain potentially relevant primary studies (last updated September 23, 2013) (Figure 1). Next, we used the same strategy to search the eight databases for potentially relevant primary studies that were published after two large previous systematic reviews on CPOE (January 1, 2007 to September 23, 2013) [4,13]. In addition, we hand-searched nine websites (AHRQ HIT Library, AHRQ PSNET, National Patient Safety Foundation, Joint Commission, Leapfrog Group, Micromedex, Institute for Healthcare Improvement), the Web of Science, and bibliographies of other publications known to us.

### Study selection

We included peer-reviewed studies, regardless of language or design, if they compared CPOE with paper-order entry and examined either of our two primary outcomes, rates of pADEs or medication errors, across a variety of clinical conditions. Eligible settings included adult medical or surgical wards, adult medical or surgical intensive care units (ICUs), emergency departments, or the entire hospital. To reduce unwarranted variability due to contextual and methodological factors, we excluded studies that were from non-hospital settings; that addressed events limited to specific conditions (for example, infections) or types of errors (for example, allergy alerts); or that compared events in highly dissimilar patient care units. As minimum criteria for study quality, we excluded studies that did not describe methods for detecting medication events, or that used incident reporting alone, which detects 0.2–-6% of events [46]. We also excluded pediatric studies because including them would increase heterogeneity: children comprise only 6% of hospitalized patients whereas ADEs disproportionately affect older adults [12,47,48].

Two investigators independently screened the article titles and then abstracts for eligibility. We obtained full-text articles when either investigator found the abstract (or title, if the abstract was unavailable) potentially eligible. Disagreements about the eligibility of full-text articles were resolved by consensus, with a third investigator participating for ties.

### Data extraction and quality assessment

We defined pADEs as injuries to patients due to medication errors. Medication errors were defined as errors in the process of prescribing, transcribing, dispensing, or administration of a medication, which had the potential to or actually did cause harm. To focus on errors involving relatively higher risk, we excluded, when reported, 'errors' described as having no or almost no potential for harm as well as incomplete or illegible orders, disallowed abbreviations, disallowed drug names, and medications given at the wrong time (see Additional file 1).

Two investigators independently extracted data from each study using a standardized form (see Additional file 1). Disagreements were resolved by consensus, with a third investigator adjudicating ties. Extracted elements included numbers of pADEs and medication errors meeting study definitions, units of exposure to risk of pADEs or medication errors (for example, number of orders, dispensed doses, admissions, or patient days). When studies reported rates or proportions rather than these elements, variance could not be estimated, so the studies could not be included in pooled effect calculations and thus we qualitatively summarized their results instead.

From the studies included in the pooled analysis of medication errors, we extracted several elements related to intervention design and implementation, context, and study methods. Elements related to intervention design included: CPOE developer (homegrown versus commercial); and presence or absence of CDSS, CDSS sophistication (basic, moderate, or advanced; see Table 1 for definitions). When information about the system developer and CDSS were missing from the published article, we contacted the original authors.

**Table 1:** Characteristics of included studies

**Reference**	**Country**	**Number of hospitals (bed size)**	**Financial status**	**Type of hospital**	**Setting in hospital**	**Baseline error rate, %**^**a**^	**Developer of CPOE system**	**Use of CPOE**	**CDSS**^**b**^	**Study design**	**Event detection methods**^**c**^
Bizovi *et al.,* 2002 [49]	USA	1 (560)	Public	Academic	ED	3.6 (visits)	Commercial (EmSTAT; CyberPlus)	Mandatory	None^d^	Pre/post	Routine pharmacist review of medication orders
Franklin *et al.,* 2009 [50-52]	UK	1 (-)	(Probably public)	Academic	General surgery ward	64.9	Commercial (ServeRx V.1:13; MDG Medical)	Mandatory	None	Pre/post	Routine pharmacist review of medication orders, medical record review, and incident reporting
Shawahna *et al.,* 2011 [53]	Pakistan	1 (1280)	Public	Academic	2 medical wards	83.8	Homegrown	Mandatory	None	Pre/post	Medical record review (R)
Shulman *et al.,* 2005 [54]	UK	1 (-)	Public	Academic	General ICU	41.1	Commercial (QS 5.6 Clinical Information System; GE Healthcare)	Not stated	None	Pre/post	Routine pharmacist review of medication orders
Leung *et al.,* 2012 [11]	USA	6 (100 to 300 each)	–	Community	Hospital-wide	42.3	Commercial (not stated)	Not stated	Present	Pre/post	Medical record and order review (B, R)
Wess *et al.,* 2007 [55]^,^[56]	USA	2 (665 and 555)	Private	Academic, Community	General surgery, Orthopedic/neurosurgical units	–	Commercial (LastWord^®^; GE, formerly IDX)	Mandatory (n = 1) and voluntary (n = 1)	Present	Pre/post	Routine pharmacist review of medication orders, with changes signed by MD
Taylor *et al.,* 2002 [57]^e^	USA	3 (1000 in total)	Private	Academic	Hospital-wide	–	Not stated	Not stated	Present	Pre/post	Quarterly review of subset of medication orders
Barron *et al.,* 2006 [58]	USA	1 (525)	Private	Academic	Hospital-wide	10.4	Homegrown	Mandatory	Basic	Pre/post	Routine pharmacist review of medication orders
Bates *et al.,* 1998 [59]	USA	1 (726)	Private	Academic	2 medical and 2 surgical wards, 2 ICUs	4.9	Homegrown	Mandatory	Basic	Pre/post	Medical record review and other means (B, R)
Van Doormal *et al.,* 2009 [60]	The Nether-lands	2 (1300 and 600)	–	Academic	2 medical wards at each hospital	99.9	Commercial (Medicator^®^; iSoft), Partly Homegrown (Theriak^®^),	Mandatory	Basic	Pre/post	Medical record and order review
Westbrook *et al.,* 2012 [61]	Australia	2 (400 and 326)	–	Academic	4 medical wards at one hospital; 1 cardiology and 1 psychiatry unit at the other hospital	99.7	Commercial (Millenium Power Orders; Cerner and MedChart; iSoft)	Exceptions allowed	Basic	Differences in differences	Routine pharmacist review of medication orders (R)
Weant *et al.,* 2007 [62]^e^	USA	1 (489)	Public	Academic	Neurosurgical ICU	–	Not stated	Not stated	Moderate^d^	Pre/post	Routine pharmacist review of medication orders, incident reporting
Bates *et al.,* 1999 [63]	USA	1 (700)	Private	Academic	2 medical wards and 1 ICU	47.3	Homegrown	Mandatory	Moderate	Pre/post	Medical record and order review plus other means
Colpaert *et al.,* 2006 [64]	Belgium	1 (-)	–	Academic	3 units within a surgical ICU	98.0	Commercial (Centricity Critical Care Clinisoft; GE Healthcare Europe)	Mandatory	Moderate	Comparison of similar units	Routine pharmacist review of medication orders (B)
Mahoney *et al.,* 2007 [65]	USA	2 (247 and 719)	Private	Academic	Hospital-wide	–	Commercial (Siemens Medical Solutions CPOE; Siemens Medical Solutions Health Services Corp)	Exceptions allowed	Moderate	Pre/post	Routine pharmacist review of medication orders, with changes accepted by MD; incident reporting
Oliven *et al.,* 2005 [66]	Israel	1 (450)	Public	Academic	Pulmonary service	62.1	Homegrown	Not stated	Moderate	Compare similar units	Medical record and order review
Igboechi *et al.,* 2003 [67]^e^	USA	1 (350)	Private	Community	Hospital-wide	–	Commercial (Ulticare System Database; Per Se Technologies	Mandatory	Moderate	Pre/post	Routine pharmacist review of medication orders
Aronsky *et al.,* 2007 [68,69]	USA	1 (658)	Private	Academic	ED	99.8 (visits)	Homegrown (WizOrder, later commercial-ized as Horizon Expert Orders; McKesson)	Not stated	Advanced^d^	Pre/post	Routine pharmacist review of medication orders
Mendendez *et al.,* 2012 [70]	Spain	1 (200)	–	Academic	Hospital-wide	5.0	Commercial (Selene; Siemens)	Not stated	Advanced ^d^	Pre/post	Trigger tool medical record review, incident reporting, and other means

CDSS, Clinical Decision Support Systems; CPOE, computerized provider order entry; ED, emergency department; ICU, intensive care unit.

^a^Percentage of hospitalizations (or emergency department visits, where noted).

^b^None = no clinical decision support system; basic = checks for drug-allergy and drug-drug interaction; moderate = basic plus at least one additional clinical decision support function; advanced = moderate plus additional capabilities [71].

^c^T = paper described training of reviewers; B = paper described blinding of reviewers to baseline versus CPOE conditions; R = Paper described methods for assessing reviewer reliability. If none of symbols appear, these were not described.

^d^Information on CDSS obtained by contacting authors.

^e^Omitted from pooled effect calculations due to lack of data related to estimating variance.

Elements related to implementation were based on an AHRQ report addressing context-sensitive patient safety practices, including CPOE. These included: factors influencing the decision to adopt, factors facilitating implementation, and aspects of implementation described in the studies, as well as timing, extent of implementation (limited number of units versus hospital-wide), and whether use was mandatory (see Additional file 1 for details) [72].

Contextual elements included setting/population (type of clinical unit within the hospital, academic status, public versus private hospital, hospital size, country, primary language in country, payer mix), and baseline proportion of hospitalizations affected by medication errors.

Methodological elements included type of study design, event detection methods, items related to study quality (adapted from relevant reporting criteria in the Standards for Quality Improvement Reporting Excellence; SQUIRE) [73], and funding source.

### Data synthesis and analysis

Using the DerSimonian–Laird random effects model [74], we conducted meta-analyses for two outcomes (pADEs and medication errors) for all eligible studies combined, and for different subgroups of studies as described below. For each eligible study and outcome measure, we calculated a risk ratio (RR) as the number of events per unit of exposure in the CPOE group divided by events per unit of exposure in the paper-order entry group. Units of exposure varied across studies. If a study provided more than one unit of exposure, we selected the unit most commonly used in the included studies.

Within each meta-analysis, we tested the heterogeneity of the log-transformed RRs using *Q* and *I*^2^ statistics [75]. Heterogeneity was present when the *I*^2^ statistic was 50% or more and the *P*-value for the Q statistic was 0.05 or less.

We conducted two sensitivity analyses, removing one study at a time from each meta-analysis to assess the influence of each individual study, and testing whether the choice of units of exposure affected results. To assess publication bias, we examined funnel plots, Begg and Mazumdar’s rank correlation test, and Egger’s regression intercept test [76].

#### ***Intervention design and implementation, contextual, and methodological factors***

*A priori*, we identified nine factors that might be associated with heterogeneity in medication errors across studies. Intervention design factors included type of CPOE developer (homegrown versus commercial), presence or absence of CDSS, and sophistication of CDSS (basic, moderate, or advanced). Intervention implementation factors included scope of implementation (hospital-wide versus limited) and timing of CPOE implementation (year CPOE was implemented or, if missing, the year the study was published). Contextual factors included country (US versus non-US) and baseline proportion of hospitalizations affected by medication errors. Methodological design factors included study design (pre-post versus other designs) and event detection methods (pharmacist order review versus more comprehensive methods). For each discrete factor, we conducted a subgroup analysis when there were at least three studies per subgroup, for example, pre/post design versus other design. For each continuous factor, we conducted a meta-regression using the factor as the sole predictor. In each meta-regression, we pooled log-transformed RRs, and presented the pooled results on the original RR scale.

Pooled meta-analyses were conducted using Comprehensive Meta-analysis, V2 (Biostat, Englewood, NJ, USA); meta-regression analyses were conducted in STATA (V13) (StataCorp LP, College Station, TX, USA).

## Results

We screened 4,891 potentially eligible records, including the bibliographies of 32 systematic reviews on CPOE or CDSS [4,5,13-42], and then examined 93 full-text articles on CPOE. Of these 93 full-text articles, 74 were excluded: 32 did not test the effectiveness of CPOE, 3 addressed non-hospital settings, 6 addressed pediatric settings, 5 used incident reporting alone to detect events, 1 did not describe event detection methods, 16 addressed outcomes other than medication errors or pADEs (for example, workflow or cost), 5 addressed errors limited to specific conditions, 1 addressed specific types of errors, 2 (1 in French) addressed errors that were excluded because they posed a lower risks of harm, 1 (in Spanish) compared event rates in dissimilar clinical units (obstetric and oncology), and 2 were duplicate publications of articles meeting the selection criteria (Figure 1; see Additional file 1, Additional file 2).

The remaining 19 original articles met the selection criteria and addressed medication errors; 7 of these also addressed pADEs (Table 1) [11,49,50,53-55,57-68,70]. Of these 19 studies, 3 omitted the data needed to estimate variance and, therefore, were excluded from pooled effect calculations, resulting in 16 eligible studies, including 6 that addressed pADEs [57,62,67].

Of the 16 studies, half were based in the US, including two in community hospitals [11,55]. Thirteen studies used pre/post designs [11,49,50,53-55,58-60,63,65,68,70], two compared similar units within a hospital during the same time period [64,66], and one compared changes over time between intervention and control units (differences in differences design) [61]. Definitions of medication errors and the methods used to detect them varied across studies (see Additional file 1). Seven studies identified events using data from routine pharmacist review of medication orders [49,54,55,58,61,64,68]. One study provided information on reviewer training [11], three on blinding of reviewers [11,59,64], and none on reliability. The baseline percentage of hospitalizations affected by medication errors ranged from 3.6% [49] to 99.9% [60].

Nine studies assessed commercially developed CPOE systems [11,49,50,54,55,61,64,65,70], six evaluated homegrown systems [53,58,59,63,66,68], and one examined both [60]. No two studies assessed the same commercial system. CDSS was present in twelve studies [11,55,58-61,63-66,68,70], and absent in four [49,50,53,54]; we contacted and obtained responses from authors for three of the studies (Table 1).

For all but one study [58], most of the desired information on implementation was missing (see Additional file 1). Based on the information that was reported, ten studies described the use of CPOE as mandatory at one or more sites [49,50,53,55,58-61,63,64]. CPOE was implemented hospital-wide in four studies [11,58,65,70], in the emergency department in two studies [49,68], and in a limited number of inpatient units in the rest. Four studies were conducted in complex organizations with facilities in multiple communities [55,59,63,65], another study was in a large hospital with affiliated clinics [49], and another was in community hospitals [11]. Past experience with information technology was reported in seven studies [49,50,55,58,59,63,65]. Three studies reported that organizational leadership influenced the adoption decision [55,58,65], and four stated that staff training and education facilitated implementation [53,54,58,66]. One study mentioned the role of staff time to learn CPOE, a person to lead implementation, extensive project management, an implementation timeline, teamwork, and patient safety culture related to CPOE [58]. Another study described the effects of having a responsible person, local tailoring, and teamwork [65].

The three studies omitted from the pooled analysis due to lack of variance estimates were similar to the included studies. They were conducted in the US in medium to large hospitals, including one in a community hospital. One study evaluated a commercially developed system [67]; the other two did not report the developer. Two studies included CDSS [57,67]. All three used pre/post designs, one detected events using pharmacist review of medication orders [67], and none reported reviewer training, blinding, or reliability. These studies also did not report implementation context or processes in detail [62,67], except for one, which discussed financial considerations and leadership [57].

### Primary outcome: preventable adverse drug events

**Figure 2:**
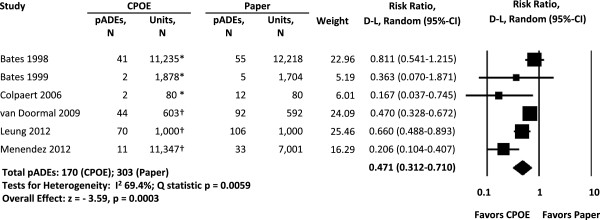
**Meta-analysis: relative risk of preventable adverse drug events using computerized provider order entry (CPOE) versus paper-order entry in hospital acute care settings.** Units of exposure: *1,000 patient days; ^†^admissions.

Of the 19 studies, 7 assessed pADEs [11,59,60,62-64,70]. For the six studies in the pooled analysis, RRs ranged from 0.17 to 0.81. Overall, CPOE was associated with about half as many pADEs as paper-order entry (pooled RR = 0.47, 95% CI 0.31 to 0.71). Studies were heterogeneous (*I*^2^ = 69%) (Figure 2). Serial removal of each study did not substantially influence results (pooled RR range 0.40 to 0.58). There was no evidence of publication bias using a funnel plot, or Begg and Mazumdar’s test (see Additional file 1). For one study excluded from the pooled analysis due to lack of data on variance, we calculated an RR of 0.11 [62].

### Secondary outcome: medication errors

**Figure 3:**
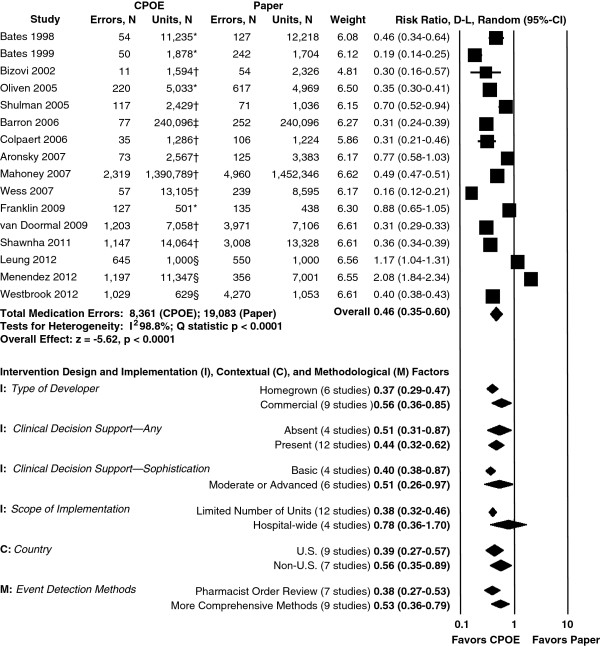
**Meta-analysis: relative risk of medication errors using computerized provider order entry (CPOE) versus paper-order entry in hospital acute care settings.** Units of exposure: *1,000 patient days; †orders; ‡dispensed doses; ^§^admissions.

All 19 studies meeting selection criteria assessed medication errors [11,49,50,53-55,57-68,70]. Across the 16 studies eligible for the pooled analysis, RRs ranged from 0.16 to 2.08. The pooled estimate showed that medication errors were approximately half as common when providers used CPOE than when they used paper-order entry (pooled RR = 0.46, 95% CI 0.35 to 0.60). The studies were highly heterogeneous (*I*^2^ = 99%) (Figure 3). Results were robust to serial removal of each individual study (pooled RR range 0.42 to 0.49), and to selection of an alternative unit of exposure in the four studies where that was possible (pooled RR = 0.45, 95% CI 0.34 to 0.59). There was no evidence of publication bias using a funnel plot, or Begg and Mazumdar’s test (see Additional file 1).

Two studies included in the pooled analysis reported increases in medication errors after the introduction of CPOE, however, both also reported statistically significant decreases in preventable adverse drug events [11,70]. A third study, excluded due to lack of data on variance, also showed an increase in errors and a decrease in pADEs, but statistical testing was not performed [62].

For two studies excluded from the pooled analysis due to lack of data on variance, we calculated RRs of 0.61 [67], and 1.73, respectively [62]. In the third such study, the authors reported a 50% decline in medication errors (see Additional file 1) [57].

### Intervention design and implementation, contextual, and methodological factors

Six of the *a priori* subgroup analyses met the requirement to have at least three studies per subgroup and were, therefore, conducted (two were on one variable, CDSS) (Figure 3). Two univariate meta-regression analyses were able to examine whether baseline medication error rate or year of publication (a proxy for maturity of CPOE intervention; date of implementation was frequently missing) predicted effectiveness.

Of five intervention design and implementation factors examined, none reached the conventional level of statistical significance, including type of developer (commercial 0.56 (95% CI 0.36 to 0.85) versus homegrown 0.37 (0.29 to 0.47)), type of CDSS (present 0.44 (0.32 to 0.62) versus absent 0.51 (0.31 to 0.87), and basic 0.40 (0.38 to 0.87) versus moderate or advanced 0.51 (0.26 to 0.97)), and scope of implementation (hospital-wide 0.78 (0.36 to 1.70) versus limited 0.38 (0.32 to 0.46)). Year of publication was not associated with differential effectiveness.

Two contextual factors were evaluated. Studies performed in the US showed greater effectiveness than non-US studies, but this difference was not statistically significant. As the baseline percentage of hospitalizations associated with medication errors increased from 3.6% to 99.9% (data available for 12 studies), the predicted RR of medication errors with CPOE decreased from 1.90 to 0.08 (*P* < 0.001).

Regarding methodological factors, studies that used pharmacist order review reported greater effectiveness than studies using more comprehensive event detection methods, although this difference was not statistically significant. Almost all studies used pre/post designs so this subgroup analysis was not conducted.

## Discussion

The principal finding of this analysis is that CPOE is associated with a significant reduction in pADEs (hat is, the patient injuries it was designed to prevent) in adult hospital-related acute care settings. Specifically, compared with using paper orders, using CPOE was associated with about half as many pADEs. Medication errors, likewise, were also about half as common with CPOE as with paper-order entry, and the reduction was generally similar across studies with different intervention designs and different implementation, contextual, and methodological characteristics. There were no statistically significant differences in effect between commercial and homegrown systems, with or without CDSS of differing sophistication levels, and between hospital-wide or more limited implementations. The baseline rate of hospitalizations associated with medication errors was significantly associated with effectiveness, as increasing baseline rates of errors were associated with increasing effectiveness. This is expected, because, with few errors, there can be little to change.

Our pooled analysis is conclusive that CPOE is associated with a reduction in pADEs. Shamliyan *et al*. examined ADEs that might or might not have been related to medication errors, and, therefore, were not as likely to be affected by CPOE. These authors observed significant declines in only three of seven studies (including pediatric ones), and did not perform a pooled analysis [37].

With regards to the overall pooled result for medication errors, our findings are generally consistent with those of earlier, more limited systematic reviews and meta-analyses [34,37,41]. Radley and colleagues also found that medication error rates declined by about half with CPOE implementation (48%, 95% CI 41 to 55%), using a small set of early studies [34]. Van Rosse and colleagues observed greater effectiveness with CPOE than we did (RR of medication errors = 0.08, 95% CI 0.01 41 to 0.76), but examined only three diverse studies [41]. Shamliyan and colleagues found that CPOE was slightly more effective than we did (odds ratio for medication errors = 0.34, 95% CI 0.22 41 to 0.52), based on inpatient and outpatient studies from before 2006 [37]. In comparison to these previous studies, we were able to identify a greater number of relevant articles despite having more restrictive selection criteria (see Additional file 1), enabling us to explore reasons for study heterogeneity.

Also like previous reviews [37], we observed substantial variability across studies in the effectiveness of CPOE at reducing medication errors. It has long been suspected that variability in the effectiveness of a complex sociotechnical intervention such as CPOE may be related not only to intervention design but also to context and implementation factors [16,77,78]. However, across the intervention design and implementation as well as contextual variables that we assessed, we did not see any statistically significant differences in the associations between CPOE use and reductions in medication errors. Two studies of commercial CPOE systems in hospital-wide implementations reported increases in medication errors but reductions in pADEs [11,70]. One potential explanation for these seemingly contradictory results is that the CPOE systems may have created new medication errors at lower risk for causing ADEs (such as concurrent submission of duplicate orders due to order sets) but reduced medication errors at higher risk of causing ADEs (such as serious drug-drug interactions). Alternatively, CPOE may have made errors easier to detect. The potential to create new types of low-risk medication errors calls attention to the importance of tailoring the CPOE system to the local environment because such errors place a time burden on providers.

This analysis has limitations. We relied on 32 previous systematic reviews to detect primary studies published before 2007. Because each review detected a slightly different set of publications (see Additional file 1), performing our own search of that period would have been unlikely to detect additional studies. We excluded pediatric studies instead of examining population age as a subgroup because these groups differ in their risk for experiencing medication errors and pADEs. Future investigators could evaluate the feasibility of conducting a similar meta-analysis for pediatric populations. We also excluded studies that relied upon incident reporting or did not describe event detection methods, considering these to be minimum criteria for study quality. The number of studies that examined pADEs was not large, but all studies detected declines. Most studies were conducted in academic centers, limiting generalizability to community hospitals. Finally, the included studies all used limited methods, including using pre/post designs and lacking robust data-collection methods.

## Conclusion

Implementing CPOE is associated with a greater than 50% decline in pADE rates in hospital-related settings, although results vary. Medication errors decline to a similar degree. Changes in medication errors appear to be consistent across commercial and homegrown systems, with or without clinical decision support, and in individual units or hospital-wide implementations. Many context and implementation variables have, unfortunately, not been reported sufficiently to assess their association with effectiveness. Overall, these findings suggest that the CPOE requirements for meaningful use under the HITECH Act may benefit public health. Knowledge about how to make CPOE more effective would be greatly facilitated by greater reporting of context and implementation details.

## Abbreviations

ADE: Adverse drug event; pADE: Preventable adverse drug event; CDSS: Clinical decision support systems; CPOE: Computerized provider order entry; ED: emergency department; EHR: Electronic health record; HITECH: Health Information Technology for Economic and Clinical Health; ICU: intensive care unit.

## Competing interests

The authors have no conflicts of interest with the work.

## Authors’ contributions

TKN, CSS, PGS: conception and design, data collection and analysis, manuscript writing; SCM data analysis and manuscript writing; SMA conception and design; VMP, LJA, and ELD: data collection and analysis. All authors read and approved the final manuscript.

## Supplementary Material

Additional file 1 Appendix. Click here for file

Additional file 2 PRISMA Checklist. Click here for file
