# Supplementary material for: The effectiveness of computerized order entry at reducing preventable adverse drug events and medication errors in hospital settings: a systematic review and meta-analysis
Source: Syst Rev. 2014 Jun 4;3:56. doi: 10.1186/2046-4053-3-56 (PMC4096499; doi:10.1186/2046-4053-3-56)
Supplement: Additional file 1 — Appendix. [file 2046-4053-3-56-S1.doc]

**Appendix**

**Factors Influencing the Effectiveness of Computerized Order Entry at Reducing Medication Errors and Adverse Drug Events in Hospital Settings: A Systematic Review and Meta-Analysis**

**Authors: Teryl K. Nuckols, MD, MSHS; Crystal Smith-Spangler, MD, MS; Sally C. Morton, PhD; Steven M. Asch, MD, MPH; Vaspaan Patel, MPH; Laura Anderson; Emily Deichsel; Paul Shekelle, MD, PhD**

**Appendix Table 1: Search Terms**

|  | **Concept** | **Terms** |
| --- | --- | --- |
|  | **Search PubMed for Articles on CPOE: (#1) OR (#2 AND #3) OR (#4 AND #5)** | |
| 1 | CPOE Systems | "order entry"[All Fields] OR "order-entry"[All Fields] OR "CPOE"[All Fields] OR "medical order entry systems"[mesh] |
| 2 | Electronic prescribing | "electronic prescribing"[All Fields] OR "e-prescribing"[All Fields] OR "electronic ordering"[All Fields] OR "computerized ordering"[All Fields] OR "computerized orders"[All Fields] OR "electronic alerts"[All Fields] OR "computer alerts"[All Fields] OR "computerized alerts"[All Fields] |
| 3 | Hospital setting | "Hospitals"[mesh] OR "Medication systems, hospital"[mesh] OR "Hospital administration"[mesh] OR "Pharmacy service, hospital"[mesh] OR "Hospital"[All Fields] OR "Hospitals"[All Fields] OR "hospitalized*"[All Fields] OR "hospitalization"[All Fields] OR "Inpatient"[All Fields] OR "Acute care"[All Fields] OR "Intensive care"[All Fields] OR "ICU"[All Fields] OR "ward"[All Fields] OR "wards"[All Fields] |
| 4 | Decision support | "drug toxicity/prevention and control"[mesh] OR "decision support"[All Fields] OR "decision-support"[All Fields] OR "decision making, computer-assisted"[mesh] OR "electronic alerts"[All Fields] OR "computer alerts"[All Fields] OR "computerized alerts"[All Fields] |
| 5 | Medication errors | "adverse drug event*"[All Fields] OR "medication error*"[All Fields] OR "medication safety"[All Fields] OR "patient safety"[All Fields] OR "medical error"[All Fields] |
|  | **Exclude Irrelevant Articles Based on Title Words: NOT (#6)** | |
| 6 | Irrelevant Title Words | “gene”[ti] OR “genome”[ti] OR “genomics”[ti] OR “genomic”[ti] OR “genetic”[ti] OR “in vitro”[ti] OR “in vivo”[ti] OR “proteomics”[ti] OR “mouse”[ti] OR “mice”[ti] OR “rat”[ti] OR “rats”[ti] OR “symposium”[ti] OR “symposia”[ti] OR “protein”[ti] OR “proteins”[ti] OR “embryonic”[ti] OR “imaging”[ti] OR “image-based”[ti] OR “plasma”[ti] OR “residential”[ti] OR “long-term care”[ti] OR “long term care”[ti] OR “nursing home”[ti] OR “skilled nursing facility”[ti] OR “recovery house” OR “organelle”[ti] OR “dental”[ti] OR “dentist”[ti] OR “dentistry”[ti] OR “screening”[ti] OR “interview”[ti] OR “primary care”[ti] OR “primary-care”[ti] OR “ambulatory care”[ti] OR “cancer*”[ti] OR “Symposium”[ti] OR “symposia”[ti] OR “consensus statement*”[ti] OR “position paper*”[ti] OR “interview*”[ti] OR “commentar*”[ti] OR “comment”[Publication Type] OR “editorial”[Publication Type] OR “fracture”[ti] OR “osteoporosis”[ti] OR “angiography”[ti] OR “helicopter”[ti] OR “psychiatr*”[ti] OR “tobramycin”[ti] OR “cell”[ti] OR “tumor”[ti] OR “orbital”[ti] OR “Hashimoto”[ti] OR “encephalopathy”[ti] OR “antibacterial”[ti] OR “nursing home”[ti] OR “letter to the editor”[ti] OR “correspondence”[ti] OR “opthal*”[ti] OR “radiotherapy”[ti] OR “d-dimer”[ti] OR “home health”[ti] OR “hospice”[ti] OR “robot*”[ti] OR “atrial”[ti] OR “transcranial”[ti] OR “alcoholism”[ti] OR “portal”[ti] OR “femur”[ti] OR “magnetic”[ti] OR “knee”[ti] OR “laser”[ti] OR “home monitor*”[ti] OR “beta”[ti] OR “cephalosporin”[ti] OR “poison”[ti] OR “in vivo”[ti] OR “proton”[ti] OR “sleep”[ti] OR “tissue”[ti] OR “fungal”[ti] OR “needle*”[ti] OR “nephrology*”[ti] OR “motor cortex”[ti] OR “nuclear”[ti] OR “amygdale”[ti] OR “osteochondritis”[ti] OR “talus”[ti] OR “dissecan*”[ti] OR “in-vivo”[ti] OR “bacteria”[ti] OR “biopsy”[ti] OR “titration”[ti] OR “cpap”[ti] OR “c-pap”[ti] OR “library”[ti] OR “librarian*”[ti] OR “cannabis”[ti] OR “Novalis”[ti] OR “camera”[ti] OR “embolism”[ti] OR “nutrition”[ti] OR “neonatal”[ti] OR “epedemi*”[ti] OR “memory”[ti] OR “genetic*”[ti] OR “liver”[ti] OR “prophylaxis”[ti] OR “rheumatol*”[ti] OR “carbon”[ti] OR “lesion*”[ti] OR “restless legs”[ti] OR “morphometry”[ti] OR “transfusion”[ti] OR “mice”[ti] OR “morpho*”[ti] OR “pneumatic”[ti] OR “pregnan*”[ti] OR “pump”[ti] OR “radiolog*”[ti] OR “ultrasound”[ti] OR “acid-based”[ti] OR “biochemistry”[ti] OR “outpatient”[ti] OR “microcomputer”[ti] OR “electrocardio*”[ti] OR “postmenopausal”[ti] OR “frontal lobe”[ti] OR “pituitary”[ti] OR “malformation”[ti] OR “acetate”[ti] OR “vessels”[ti] OR “rat”[ti] OR “cranio*”[ti] OR “residential”[ti] OR “injury”[ti] OR “postmortem”[ti] OR “bone mass”[ti] OR “spermatozoa”[ti] OR “dental”[ti] OR “dentist*”[ti] OR “heart rate”[ti] OR “cervix”[ti] OR “sperm”[ti] OR “cesarean”[ti] OR “infrared”[ti] OR “depression”[ti] OR “suicide”[ti] OR “cardiologist”[ti] OR “ambulatory”[ti] OR “dietary”[ti] OR “dietician”[ti] OR “diet”[ti] OR “mood”[ti] OR “aggression”[ti] OR “implant*”[ti] |
|  | **Identify Systematic Reviews: AND (#7)** | |
| 7 | Reviews | “review”[ti] OR "systematic review*"[tiab] OR "health technology assessment"[tiab] OR “metaanalysis”[tiab] OR "meta-analysis"[tiab] OR “review”[Publication Type] |

**Definitions of Medication Errors Used in the Meta-Analysis**

**Medication errors** (MEs) are errors in the process of ordering, dispensing, or administering a medication, regardless of whether an injury occurred. We included medication errors that had a potential to harm patients if they were not intercepted.

We excluded any errors that studies described as having no potential for causing harm, or that we judged to involve a relatively low probability of causing harm. We excluded events when the “error” involved an illegible order, an illegible signature, information missing from the order, trailing zeros or no leading zeros, abbreviations disallowed by the Joint Commission, or medications prescribed using a drug name other than the approved name. Including these events could overestimate the benefits of CPOE, since only a small percentage would be likely to result in patient injury yet basic CPOE features could almost completely eliminate them. We also excluded errors that involved medications being given at the wrong time due to their relatively low risk of causing harm.

Many studies detected events through routine pharmacist review of medication prescriptions, which means that most of the errors detected would be errors in the process of ordering medication. As long as the methods of detection were identical in the paper-based and computer-based ordering periods, we accepted these methods of detection.

Unlike Bates (1998), we did not exclude “intercepted” medication errors, which were reported in Bates 1999 and Leung 2012. Our rationale was that many studies used pharmacist review as a means of detecting medication errors, which is essentially a form of intercepting medication errors. Excluding “intercepted” medication errors would, therefore, reduce comparability with other studies by restricting the number of medication errors being examined and excluding errors that other studies were including. Further, the types of errors that are intercepted at any point in time by pharmacists, nurses, or other clinicians would likely have some randomness to it and, therefore, excluding them would eliminate events that might actually be preventable using CPOE-CDS.

**Preventable adverse drug events** (pADEs) are injuries due to medication errors. Preventable adverse drug events are defined by the presence of some sort of harm or injury due to a medication error. The harm need not be severe, symptomatic, or visible. For example, a laboratory value such as a PTT greater than 100 or a blood glucose level less than 40 without symptoms can be considered “harm” in some studies. We considered only preventable adverse drug events, excluding any non-preventable adverse drug events since these could not be prevented through CPOE.

We excluded any **non-preventable ADEs** reported in the studies because they do not involve medication errors and, therefore, are less likely to be preventable through the use of CPOE-CDS.

**Methods Used to Extract Information Related to Study Quality and CPOE Implementation**

To assess the quality of the studies and their reporting, we extracted information related to specific elements from the Standards for QUality Improvement Reporting Excellence (SQUIRE)[[1]](#footnote-2). Reviewers determined: study design; data collection methods (event definitions, data sources, qualifications and training of reviewers identifying events, blinding, reliability assessment), comparability of populations exposed to CPOE and paper order entry, comparability of event detection methods for CPOE and paper ordering groups, any risk adjustment methods, and funding (source, editorial independence). In addition, we drew from recommendations in the AHRQ report *Assessing the Evidence for Context-Sensitive Effectiveness and Safety of Patient Safety Practices* to develop questions related to: organizational complexity (e.g., freestanding hospital vs. part of network), existing quality/safety infrastructure and culture, past experiences with health information technology, how the external environment (e.g., regulatory requirements or incentives) and organizational leadership influenced the decision to adopt CPOE, education and training of staff in CPOE, whether an internal or external “champion” oversaw implementation, whether there was a helpdesk for users, whether project management and timelines were used to facilitate implementation, teamwork relating to CPOE implementation, whether incentives were used internally to promote implementation, how CPOE was implemented (mandatory vs. voluntary), whether implementation was an iterative process or tailored to the local environment, and whether implementation proceeded as originally planned.[[2]](#footnote-3)

**Appendix Table 2: Results of Literature Search: Original Articles on CPOE Selected for Review of Full Text Article or Identified from A Previous Systematic Review: Included and Excluded Studies, with Reasons for Exclusion**

| **Author and Year** | **Source of Article** | **Included in Meta-Analysis** | **Reasons for Exclusion** | | | | | | |
| --- | --- | --- | --- | --- | --- | --- | --- | --- | --- |
|  |  |  | **Miscellan-eous** | **Non-Hospital Setting** | **Pediatric Setting** | **Did Not Test Intervention** | **Outcome Other Than MEs or pADEs*** | **MEs, Specific Condition or Specific Error** | **Event Detection (Only Incident Reporting or Unknown)** |
| Aarts 2007 | Literature search |  |  |  |  | X |  |  |  |
| Abookire 2000 | Eslami, Chaudhry, Shekelle |  |  |  |  |  |  | X |  |
| Abramson 2012 | Literature search |  |  | X |  |  |  |  |  |
| Adams 2011 | Jones |  |  |  | X |  |  |  |  |
| Ageno 1998 | Pearson, Durieaux |  |  |  |  |  |  | X |  |
| Ageno 2000 | Pearson |  |  |  |  |  |  | X |  |
| Agrawal 2009 | Literature search |  |  |  |  | X |  |  |  |
| Al-Dorzi 2011 | Literature search |  |  |  |  |  | X |  |  |
| Ali 2005 | Eslami |  |  |  |  |  |  | X |  |
| Ali 2010 | Literature search, Manias, Jones |  | MEs with low risk of harm |  |  |  |  |  |  |
| Amarasingham 2009 | Buntin |  |  |  |  |  | X |  |  |
| Anderson 2002 | Literature search (hand search) |  |  |  |  | X |  |  |  |
| Anglim 1997 | Oren |  |  |  |  |  |  | X |  |
| Ansari 2003 | Hemens, Pearson |  |  | X |  |  |  |  |  |
| Apkon 2005 | Pearson |  |  | X |  |  |  |  |  |
| Appari 2011 | Jones |  |  |  |  |  | X |  |  |
| ***Aronsky 2007*** | Literature search | Included |  |  |  |  |  |  |  |
| Asaro 2006 | Eslami, Weir, Goldzweig |  |  |  |  |  |  | X |  |
| Asaro 2008 | Hand search, Buntin |  |  |  |  |  | X |  |  |
| Ash 1999 | Hand search |  |  |  |  | X |  |  |  |
| Ash 2007 | Hand search |  |  |  |  | X |  |  |  |
| Ash 2009 | Buntin |  |  |  |  |  | X |  |  |
| Atreja 2005 | Kuperman |  |  | X |  |  |  |  |  |
| Banet 2006 | Eslami |  |  |  |  |  | X |  |  |
| ***Barron 2006*** | Weir | Included |  |  |  |  |  |  |  |
| Bates 1994 | Eslami, Shekelle, Oren |  |  |  |  |  | X |  |  |
| ***Bates 1998*** | Ammenwerth, Eslami, Weir, Wolfstadt, Schedlbauer, Shamliyan, Maslove, Chaudhry, Oren, Colpaert 2009 | Included |  |  |  |  |  |  |  |
| ***Bates 1999 JAMIA*** | Ammenwerth, Eslami, Weir, Wolfstadt, Schedlbauer, Shamliyan, Kuperman, Maslove, Chaudhry, Oren, Radley, Kawamoto, Colpaert 2009 | Included |  |  |  |  |  |  |  |
| Bates 1999 Am J Med | Bright, Kuperman, Shojania 2010 |  |  |  |  |  | X |  |  |
| Begg 1989 | Durieaux |  |  |  |  |  |  | X |  |
| Berner 2006 | Mollon, McKibbon |  |  | X |  |  |  |  |  |
| Bertoni 2009 | Hemens, McKibbon |  |  | X |  |  |  |  |  |
| Beuscart-Zephir 2005 | Eslami |  |  |  |  |  | X |  |  |
| ***Bizovi 2002*** | Ammenwerth, Eslami, Weir, Shamliyan, Radley | Included |  |  |  |  |  |  |  |
| Blankenship 2012 | Literature search, Jones |  |  |  |  |  | X |  |  |
| Bloomfield 2005 | Pearson, McKibbon |  |  | X |  |  |  |  |  |
| Bogucki 2004 | Eslami |  |  |  |  |  |  | X |  |
| Bomba 2006 | Eslami |  |  |  |  |  | X |  |  |
| Bradley VM 2006 | Literature search, Manias |  |  |  |  |  |  |  | Reporting |
| Buising 2008 | Buntin |  |  |  |  |  | X |  |  |
| Burton 1991 | Durieaux, Shekelle, Pearson |  |  |  |  |  |  | X |  |
| Butler 2006 | Eslami, Weir, Goldzweig |  |  |  |  |  |  | X |  |
| Campbell 2006 | Weir 2012 |  |  |  |  | X |  |  |  |
| Cannon 2000 | Jamal |  |  |  |  |  |  | X |  |
| Cartmill 2012 | Jones |  |  |  |  |  | X |  |  |
| Casner 1993 | Pearson, Durieaux |  |  |  |  |  | X |  |  |
| Chen 2011 | Jones |  |  |  | X |  |  |  |  |
| Cheng 2003 | Eslami |  |  |  |  |  | X |  |  |
| Cheng 2012 | Jones |  |  |  |  |  |  | X |  |
| Chertow 2001 | Ammenwerth, Eslami, Kuperman, Chaudhry, Jamal, Durieaux |  |  |  |  |  |  | X |  |
| Chiarelli 1990 | Shekelle |  |  |  | X |  |  | X |  |
| Chin 1995 | Weir |  |  | X |  |  |  |  |  |
| Chin 1999 | Chaudhry, Shekelle |  |  |  |  |  |  | X |  |
| Chisolm 2006 | Eslami |  |  |  |  |  |  | X |  |
| Christakis 2001 | Hemens, McKibbon, Shojania 2009, Shojania 2010, Kawamoto |  |  | X |  |  |  |  |  |
| Clemens 2011 | Jones |  |  |  |  |  | X |  |  |
| Cobos 2005 | Hemens, McKibbon, Pearson |  |  | X |  |  |  |  |  |
| Coe 1977 | Hemens |  |  | X |  |  | X |  |  |
| Collins 2004 | Weir, Buntin |  |  |  |  |  |  | X |  |
| Collins 2007 | Buntin |  |  |  |  |  | X |  |  |
| Collins 2011 | Literature search |  |  |  |  |  |  | X |  |
| Colon-Emeric 2009 | Buntin |  |  | X |  |  | X |  |  |
| ***Colpaert 2006*** | Ammenwerth, Weir, Wolfstadt, Reckmann, Maslove, van Rosse, McKibbon, Colpaert 2009 | Included |  |  |  |  |  |  |  |
| Cook 2011 | Jones |  |  |  |  |  | X |  |  |
| Cordero 2004 | Ammenwerth, Eslami, Weir, Shamliyan, Maslove, Chaudhry, van Rosse , Shekelle, Radley |  |  |  | X |  |  |  |  |
| **C**osta 2004 | Eslami |  |  |  |  | X |  |  |  |
| Daniels 2012 | Literature search, Jones |  |  |  |  |  |  | X |  |
| Daurio 2009 | Buntin |  |  |  |  |  | X |  |  |
| Davis 2007 | Pearson, Hemens, McKibbon |  |  |  | X |  |  |  |  |
| Davis 2009 | Buntin |  |  | X |  |  | X |  |  |
| Del Beccaro 2006 | Eslami, Weir, Goldzweig, van Rosse, Maslove |  |  |  | X |  |  |  |  |
| Del Fiol 2000 | Eslami |  |  |  |  |  |  | X |  |
| Derose 2005 | Hemens, Nies |  |  |  |  |  | X |  |  |
| Destache 1990 | Durieaux |  |  |  |  |  |  | X |  |
| Devine 2010 | Jones |  |  | X |  |  |  |  |  |
| Dexheimer 2008 | Buntin |  |  |  |  |  | X |  |  |
| Dexter 1998 | Chaudhry |  |  |  |  |  |  | X |  |
| Dexter 2001 | Eslami, Goldzweig, Mollon, McKibbon, Shojania 2009, Shojania 2010, Pearson |  |  |  |  |  | X |  |  |
| Dexter 2004 | Eslami, Chaudhry, Jamal |  |  |  |  |  |  | X |  |
| Diana 2011 | Literature Search |  |  |  |  | X |  |  |  |
| Di Pentima 2009 | Buntin |  |  |  |  | X |  | X |  |
| Donyai 2007 | Reckmann |  | Duplicate Publication (Franklin 2009) |  |  |  |  |  |  |
| DuBeshter 2006 | Eslami |  |  |  |  |  |  | X |  |
| Durieaux 2000 | Jamal, Literature Search—Cochrane Library |  |  |  |  |  |  | X |  |
| Eccles 2002 | Jamal, Shojania 2009, Shojania 2010, Pearson |  |  | X |  |  |  | X |  |
| Effken 2009 | Literature Search – Hand Search |  |  |  |  | X |  |  |  |
| Eppenga 2011 | Literature Search |  |  |  |  | X |  |  |  |
| Eslami 2006 | Eslami |  |  |  |  |  |  | X |  |
| Evans, 1994 | Wolfstadt, Chaudhry, McKibbon |  |  |  |  |  |  | X |  |
| Evans, 1995 | Wolfstadt |  |  |  |  |  |  | X |  |
| Evans 1998 NEJM | Ammenwerth, Eslami, Wolfstadt, Maslove, Jamal, Radley, Oren, Wolfstadt |  |  |  |  |  |  | X |  |
| Evans 1998 Critical Care | Weir, Reckmann, Oren, Manias |  |  |  |  |  | X |  |  |
| Evans 1999 | Eslami, Chaudhry |  |  |  |  |  |  | X |  |
| Fair 2004 | Kuperman |  |  |  |  | X |  |  |  |
| Feldman 2013 | Jones |  |  |  |  |  | X |  |  |
| Feldstein 2006 | Hemens |  |  | X |  |  |  |  |  |
| Feldstein 2006 | Pearson |  |  | X |  |  |  | X |  |
| Feldstein 2006 | Pearson |  |  |  |  |  |  | X |  |
| Feldstein 2006 | Ammenwerth, Pearson |  |  |  |  |  |  | X |  |
| Fernandez 2007 | Maslove |  |  |  |  |  |  | X |  |
| Fernandez Perez 2007 | Buntin, Maslove |  |  |  |  |  | X |  |  |
| Fernando 2012 | Jones |  |  |  |  | X |  |  |  |
| Field 2009 | McKibbon, Hemens |  |  | X |  |  |  | X |  |
| Fihn 1994 | Chaudhry, Pearson, Bright |  |  |  |  |  |  | X |  |
| Filippi 2003 | Shojania 2009, Shojania 2003, Hemens, McKibbon |  |  | X |  |  |  | X |  |
| Fisher 2003 | Eslami, Kuperman |  |  |  |  |  | X |  |  |
| FitzHenry 2011 | Jones |  |  |  |  | X |  |  |  |
| Fitzmaurice 2000 | Durieaux |  |  |  |  |  |  | X |  |
| Flannagan 1999 | Kawamoto |  |  | X |  |  |  |  |  |
| Flottorp 2002 | Shojania 2009, Shojania 2010, Pearson, McKibbon |  |  |  |  |  |  | X |  |
| Fontan 2003 | Weir, Shamliyan |  |  |  | X |  |  | X |  |
| Fortuna 2009 | Hemens, McKibbon |  |  |  |  |  | X |  |  |
| Fraenkel 2003 | Ammenwerth, Maslove |  |  |  |  |  |  |  | Reporting |
| Frank 2004 | Shojania 2009, Shojania 2010, McKibbon |  |  | X |  |  |  |  |  |
| Franklin 2007 | Reckmann |  | Duplicate Publication (Franklin 2009) |  |  |  |  |  |  |
| ***Franklin 2009*** | Literature Search – hand search | Included |  |  |  |  |  |  |  |
| Furukawa 2010 | Jones |  |  |  |  | X |  |  |  |
| Gagnon 2006 | Literature Search – hand search |  |  |  |  | X |  |  |  |
| Galanter 2004 | Goldzweig, , Schedlbauer |  |  |  |  |  |  | X |  |
| Galanter 2005 | Ammenwerth, Eslami |  |  |  |  |  |  | X |  |
| Galanter 2013 | Jones |  |  |  |  |  |  | X |  |
| Gandhi 2005 | Ammenwerth, Weir, Shamliyan |  |  | X |  |  |  |  |  |
| Garrett 2008 | Buntin |  |  |  |  |  | X |  |  |
| Garrido 2005 | Goldzweig |  |  | X |  |  |  |  |  |
| Georgiou 2007 | Buntin |  |  |  |  |  | X |  |  |
| Georgiou 2009 | Buntin |  |  |  |  |  | X |  |  |
| Georgiou 2011 | Jones |  |  |  |  |  | X |  |  |
| Ghahramani 2009 | Buntin |  |  |  |  |  | X |  |  |
| Gilutz 2009 | Hemens, McKibbon |  |  | X |  |  | X |  |  |
| Glemaud 2000 | Oren |  |  |  |  |  |  | X |  |
| Gonzalez 1989 | Durieaux |  |  |  |  |  |  | X |  |
| Griffey 2011 | Literature search |  |  |  |  |  | X (ADEs) |  |  |
| Graumlich 2009 | McKibbon, Bright |  |  | X |  |  |  |  |  |
| Guerra 2010 | Jones |  |  |  |  |  | X |  |  |
| Gurwitz 2008 | Literature search, Bright, Hemens |  |  | X |  |  |  |  |  |
| Han 2005 | Eslami, Weir, Goldzweig, van Rosse, Maslove |  |  |  | X |  |  |  |  |
| Harpole 1997 | Kawamoto |  |  |  |  |  | X |  |  |
| Haynes 2011 | Literature search, Jones |  |  |  |  |  | X | X |  |
| Heidenreich 2005 | Hemens |  |  |  |  |  | X |  |  |
| Heidenreich 2007 | Hemens |  |  |  |  |  | X |  |  |
| Hensing 2008 | Buntin |  |  |  |  |  | X |  |  |
| Henstrom 2007 | Georgiou |  |  |  |  |  | X |  |  |
| Hetlevik 1999 | McKibbon |  |  | X |  |  |  |  |  |
| Hetletvik 2000 | Jamal |  |  |  |  |  |  | X |  |
| Hicks 2008 | Shojania 2009, Shojania 2010, Hemens, McKibbon |  |  |  |  |  |  | X |  |
| Hickling 1989 | Durieaux |  |  |  |  |  |  | X |  |
| Hill 2010 | Jones |  |  |  |  | X |  |  |  |
| Himmelstein 2010 | Buntin |  |  |  |  |  | X |  |  |
| Hinson 1993 | Literature Search – hand search |  | Not on CPOE |  |  |  |  |  |  |
| Holden 2010 | Buntin |  |  |  |  |  | X |  |  |
| Holden 2012 | Jones |  |  |  |  | X |  |  |  |
| Holdsworth 2007 | Weir, van Rosse |  |  |  | X |  |  |  |  |
| Hoonakker 2012 | Jones |  |  |  |  |  | X |  |  |
| Horskey 2005 | Eslami |  |  |  |  |  |  | X |  |
| Horning 2011 | Literature search |  |  |  |  | X |  |  |  |
| Hsieh 2004 | Eslami |  |  |  |  |  |  | X |  |
| Hulgan 2004 | Eslami, Pearson |  |  |  |  |  |  | X |  |
| Hurley 1986 | Durieaux |  |  |  |  |  |  |  |  |
| Hwang 2002 | Literature search |  |  |  |  |  | X |  |  |
| Hyman 2012 | Jones |  |  |  |  | X |  |  |  |
| ***Igboechi 2003*** | Ammenwerth, Eslami, Kuperman | Omitted, no variance info |  |  |  |  |  |  |  |
| Jani 2011 | Literature search |  |  |  | X |  |  |  |  |
| Jaspers 2008 | Buntin |  |  |  |  |  | X |  |  |
| Javitt 2005 | Mollon, Hemens, McKibbon |  |  |  |  |  | X |  |  |
| Jayawardena 2007 | Literature search |  |  |  |  | X |  |  |  |
| Jensen 2006 | Weir |  |  |  |  |  | X |  |  |
| Jha 2008 | Buntin |  |  |  |  | X |  |  |  |
| Jiménez Muñoz 2011 | Literature search |  | Compared Dissimilar Clinical Units |  |  |  |  |  |  |
| Jones 2011 | Jones |  |  |  |  | X |  |  |  |
| Judge 2006 | Eslami, Mollon, Shojania 2009, Shojania 2010, Pearson, Hemens |  |  | X |  |  |  |  |  |
| Kattan 2006 | Hemens |  |  | X |  |  |  |  |  |
| Kaushal 2006 | Eslami |  |  |  |  |  | X |  |  |
| Kawahara 1989 | Eslami |  |  |  |  |  |  | X |  |
| Keene 2007 | Weir, Maslove, van Rosse |  |  |  | X |  | X |  |  |
| Kenealy 2005 | Shojania 2009, Shojania 2010 |  |  | X |  |  |  | X |  |
| Keshavjee 2001 | Weir |  |  | X |  |  | X |  |  |
| Kho 2008 | Buntin |  |  |  |  |  | X |  |  |
| Kilgore 1998 | Chaudhry |  |  |  |  |  | X |  |  |
| Kim 2006 | Ammenwerth, Eslami, Weir, Reckmann, Radley |  |  |  | X |  |  |  |  |
| King 2003 | Ammenwerth, Eslami, Weir, Shamliyan, van Rosse |  |  |  | X |  |  |  |  |
| Kinn 2002 | Weir |  |  | X |  |  |  |  |  |
| Kirk 2005 | Ammenwerth |  |  |  | X |  |  |  |  |
| Ko 2007 | Literature search |  |  |  |  | X |  |  |  |
| Koide 2000 | Ammenwerth, Pearson |  |  |  |  |  |  | X |  |
| Koppel 2005 | Eslami, Kuperman, Maslove |  |  |  |  |  | X |  |  |
| Kralj 2003 | Shojania 2009, Shojania 2010, Pearson |  |  | X |  |  |  |  |  |
| Krall 2004 | Hemens, McKibbon, Shojania 2010 |  |  | X |  |  |  |  |  |
| Krampera 2004 | Eslami |  |  |  |  |  |  | X |  |
| Kucher 2005 | Chaudhry, Shojania 2009, Shojania 2010, Pearson, Shekelle, Nies |  |  |  |  |  |  | X |  |
| Kuilboer 2006 | Pearson, McKibbon, Hemens |  |  |  |  |  |  | X |  |
| Kuperman 1996 | Chaudhry |  |  |  |  |  |  | X |  |
| Kuperman 1999 | Kawamoto |  |  |  |  |  | X |  |  |
| Lacasa 2012 | Literature search |  |  |  |  | X |  |  |  |
| Larsen 1989 | Chaudhry |  |  |  |  |  |  | X |  |
| Lee 1996 | Eslami |  |  |  |  | X |  |  |  |
| Lehman 2001 | Weir |  |  |  |  |  | X |  |  |
| Lesourd 2002 | Pearson, Durieaux |  |  |  |  |  |  | X |  |
| Lester 2006 | Pearson, Hemens |  |  |  |  |  |  | X |  |
| ***Leung 2012*** | Literature search, Jones | Included |  |  |  |  |  |  |  |
| Litvin 2013 | Jones |  |  |  |  |  | X |  |  |
| Litzelman 1993 | Chaudhry |  |  |  |  |  |  | X |  |
| Lo 2009 | Hemens, McKibbon |  |  | X |  |  |  |  |  |
| Longhurst 2010 | Jones |  |  |  |  |  | X |  |  |
| Mack 2009 | Maslove |  |  |  | X |  |  |  |  |
| Madaras-Kelly 2006 | Pearson |  |  |  |  |  |  | X |  |
| Magrabi 2011 | Literature search |  |  |  |  | X |  |  |  |
| ***Mahoney 2007*** | Reckmann | Included |  |  |  |  |  |  |  |
| Makoul 2001 | Weir |  |  | X |  |  |  |  |  |
| Manjoney 2004 | Literature Search – hand search |  |  |  |  | X |  |  |  |
| Manotti 2001 | Pearson, Durieaux |  |  |  |  |  |  | X |  |
| Manzo 2005 | Eslami |  |  |  |  |  |  |  | Unclear |
| Martens 2007 | Mollon, Pearson, Buntin, Hemens, McKibbon |  |  | X |  |  |  |  |  |
| Martens 2008 | Buntin |  |  | X |  |  |  |  |  |
| Matheny 2008 | Hemens, McKibbon |  |  | X |  |  |  |  |  |
| Mattison 2010 | Jones |  |  |  |  |  | X |  |  |
| Maurer 2003 | Ammenwerth |  | Studied MEs, Excluded from Study |  |  |  |  |  |  |
| Mayer 2010 | Jones |  |  |  |  |  | X |  |  |
| Mazars 2012 | Jones |  |  |  | X |  |  |  |  |
| McAlearney 2006 | Eslami |  |  |  |  | X |  |  |  |
| McCluggage 2010 | Jones |  |  |  |  |  | X |  |  |
| McCowan 2001 | Shojania 2009, Shojania 2010, Pearson |  |  |  |  |  |  | X |  |
| McCoy 2010 | Jones |  |  |  |  |  |  |  |  |
| McDonald 1976 | Hemens, McKibbon |  |  | X |  |  |  |  |  |
| McDonald 1980 | Mollon, Chaudhry, Hemens |  |  | X |  |  |  |  |  |
| McDonald 1992 | Chaudhry |  |  |  |  |  |  | X |  |
| McGregor 2006 | McKibbon, Bright |  |  |  |  |  | X |  |  |
| McMullin 2004 | Weir |  |  | X |  |  |  |  |  |
| McMullin 2005 | Weir |  |  | X |  |  |  |  |  |
| Meigs 2003 | Shojania 2009, Shojania 2010, McKibbon |  |  |  |  |  |  | X |  |
| Mekhjian 2002 | Eslami, Weir, Chaudhry |  |  |  |  |  | X |  |  |
| Menachemi 2011 | Literature search |  |  |  |  | X |  |  |  |
| ***Menendez 2012*** | Literature search | Included |  |  |  |  |  |  |  |
| Metzger 2010 | Jones |  |  |  |  | X |  |  |  |
| Milani 2011 | Literature search |  |  |  |  |  |  | X |  |
| Milani 2012 | Jones |  |  |  |  |  | X |  |  |
| Mirco 2005 | Eslami, Weir |  |  |  |  | X |  |  |  |
| Mitchell 2004 | Ammenwerth, Eslami |  |  |  |  | X |  |  |  |
| Mitra 2005 | Pearson, Nies |  |  |  |  |  |  | X |  |
| Montgomery 2000 | Pearson, McKibbon |  |  | X |  |  |  | X |  |
| Mullett 2001 | Ammenwerth, Eslami, Wolfstadt, Maslove, Chaudhry, Shekelle |  |  |  |  |  |  | X |  |
| Mungall 1994 | Durieaux |  |  |  |  |  |  | X |  |
| Murray 2004 | Goldzweig, Pearson, Hemens, McKibbon |  |  |  |  |  |  | X |  |
| Murff 2001 | Eslami |  |  |  |  |  | X |  |  |
| Muzyk 2012 | Jones |  |  |  |  |  | X |  |  |
| Nebeker 2005 | Eslami, Kuperman |  |  |  |  | X |  |  |  |
| Niazkhani 2008a | Buntin |  |  |  |  |  | X |  |  |
| Niazkhani 2008b | Buntin |  |  |  |  |  | X |  |  |
| Niazkhani 2009 | Buntin |  |  |  |  |  | X |  |  |
| Nightingale 2000 | Eslami, Kuperman |  |  |  |  | X |  | X |  |
| ***Oliven 2005*** | Ammenwerth, Weir, Reckmann, shamliyan | Included |  |  |  |  |  |  |  |
| Ornstein 1995 | Shekelle |  |  |  |  |  |  | X |  |
| Oppenheim 2002 | Eslami |  |  |  |  |  |  | X |  |
| Ostbye 1997 | Weir |  |  |  |  |  | X |  |  |
| Overhage 1996 | Eslami, Chaudhry, Shojania 2009, Shojania 2010, Pearson, McKibbon, Kawamoto |  |  |  |  |  |  | X |  |
| Overhage 1997 Jamia | Eslami, Mollon, Kuperman, Chaudhry, Shojania 2009, Shojania 2010, Pearson, Oren, Kawamoto |  |  |  |  | X |  |  |  |
| Overhage 2001 | Weir, Shekelle, Oren, McKibbon |  |  | X |  |  | X |  |  |
| Ozdas 2006 | Eslami, Goldzweig |  |  |  |  |  |  | X |  |
| Palchuk 2005 | Kuperman |  |  | X |  |  |  |  |  |
| Palen 2006 | Goldzweig, Pearson, Hemens, McKibbon |  |  | X |  |  |  |  |  |
| Paul 2006 | Pearson, Hemens, McKibbon, Bright |  |  |  |  |  |  | X |  |
| Pestotnik 1996 | Ammenwerth |  |  |  |  |  |  | X |  |
| Peterson 2005 | Ammenwerth, Eslami, Wolfstadt, Schedlbauer |  |  |  |  |  |  | X |  |
| Peterson 2007 | Shojania 2009, Shojania 2010, Pearson, Hemens, McKibbon |  |  |  |  |  |  | X |  |
| Piazza 2009 | Buntin |  |  |  |  |  | X |  |  |
| Pillemer 2011 | Jones |  |  | X |  |  |  |  |  |
| Pirnejad 2008 | Buntin |  |  |  |  |  | X |  |  |
| Pirnejad 2009 | Buntin |  |  |  |  |  | X |  |  |
| Pizziferri 2005 | Weir, Chaudhry |  |  | X |  |  | X |  |  |
| Plaza 2005 | Hemens, McKibbon |  |  | X |  |  |  |  |  |
| Poller 1998 | Pearson, Durieaux |  |  |  |  |  |  | X |  |
| Potts 2004 | Ammenwerth, Eslami, Weir, Goldzweig, Reckmann, Shamliyan, Maslove, Chaudhry, van Rosse , Shekelle |  |  |  | X |  |  |  |  |
| Quinn 2008 | Hemens, McKibbon |  |  |  |  |  | X |  |  |
| Raebel 2005 | Hemens, McKibbon |  |  | X |  |  |  |  |  |
| Raebel 2007 | Mollon, Hemens, McKibbon |  |  |  |  |  |  | X |  |
| Raebel 2007 | Hemens, McKibbon |  |  | X |  |  |  |  |  |
| Reeve 2008 | Hemens, McKibbon |  |  | X |  |  |  |  |  |
| Refuerzo 2011 | Literature search, Jones |  |  |  |  |  | X |  |  |
| Rind 1994 | Kaushal 2003, Schedlbauer |  |  |  |  |  |  | X |  |
| Roberts 2010 | Jones |  | Paper and CPOE groups used different event detection methods |  |  |  |  |  |  |
| Rodman 1984 | Durieaux |  |  |  |  |  |  | X |  |
| Rollman 2001 | Chaudhry |  |  | X |  |  |  |  |  |
| Rollman 2002 | Chaudhry, Mollon |  |  | X |  |  |  |  |  |
| Rommers 2011 | Literature search |  |  |  |  | X |  |  |  |
| Rosenbloom 2004 | Eslami |  |  |  |  | X |  |  |  |
| Rosenbloom 2005 | Eslami, McKibbon |  |  |  |  |  | X |  |  |
| Rossi 1997 | Hemens, Mollon |  |  | X |  |  |  |  |  |
| Rothschild 2007 | Goldzweig, Shojania 2009, Shojania 2010 |  |  |  |  |  |  | X |  |
| Rotman 1996 | Mollon, Hemens, Kawamoto |  |  | X |  |  |  |  |  |
| Roumie 2006 | Shojania 2009, Shojania 2010, Pearson, McKibbon, Bright |  |  |  |  |  |  | X |  |
| Ruiz 1993 | Durieaux |  |  |  |  |  |  | X |  |
| Safran 1995 | Shojania 2010 |  |  |  |  |  | X |  |  |
| Samore 2005 JAMA | Mollon |  |  |  |  |  | X |  |  |
| Scott 2011 | Literature search |  |  |  |  | X |  |  |  |
| Sequist 2005 | Shojania 2009, Shojania 2010, Pearson, Hemens, McKibbon |  |  |  |  |  |  | X |  |
| Seidling 2010 | Literature search |  |  |  |  | X |  |  |  |
| [***Shawahna 2011***](http://www.ncbi.nlm.nih.gov/pubmed?term="Shawahna R"%5BAuthor%5D) | Literature search | Included |  |  |  |  |  |  |  |
| Shojania 1998 | Eslami, Kuperman, Chaudhry, Pearson, Oren, McKibbon |  |  |  |  |  |  | X |  |
| Shu 2001 | Eslami, Weir |  |  |  |  |  | X |  |  |
| ***Shulman 2005*** | Ammenwerth, Eslami, Weir, Reckmann, Shamliyan, Maslove, van Rosse, Colpaert 2009 | Included |  |  |  |  |  |  |  |
| Smith 2006 | Pearson |  |  | X |  |  |  |  |  |
| Smith 2006 | Goldzweig |  |  | X |  |  |  |  |  |
| Spaulding 2011 | Literature search, Jones |  |  |  |  |  | X |  |  |
| Spencer 2005 | Ammenwerth, Weir, Reckmann |  |  |  |  |  |  |  | Reporting |
| Spetz 2009 | Buntin |  |  |  |  |  |  |  | Reporting |
| Stair 1995 | Weir |  |  |  |  |  | X |  |  |
| Steele 1989 | Chaudhry |  |  | X |  |  |  |  |  |
| Steele 2005 | Schedlbauer, Wolfstadt |  |  | X |  |  |  |  |  |
| Stone 2009 | Literature search, Buntin |  |  |  |  |  |  |  | Reporting |
| Strom 2010 JAMIA | Jones |  |  |  |  |  | X |  |  |
| Strom 2010 Arch Intern Med | Jones |  |  |  |  |  | X |  |  |
| Sulmasy 1998 | Weir |  |  |  |  |  |  | X |  |
| Szilagyi 1992 | Shekelle |  |  |  | X |  |  |  |  |
| Taegtmeyer 2011 | Jones |  |  |  |  |  | X |  |  |
| Tamblyn 2003 | Ammenwerth, Mollon, Shojania 2009, Shojania 2010, Pearson, Hemens, McKibbon |  |  | X |  |  |  |  |  |
| Tan 2009 | Literature search—Cochrane Library |  |  |  | X |  |  |  |  |
| Tang 1999 | Weir, Pearson |  |  | X |  |  |  |  |  |
| Tape 1993 | Shojania 2010 |  |  | X |  |  |  |  |  |
| ***Taylor 2002*** | Eslami | Omitted, no variance info |  |  |  |  |  |  |  |
| Taylor 2008 | Radley |  |  |  | X |  |  |  |  |
| Teich 2000 | Ammenwerth, Eslami, Weir, Schedlbauer, Kuperman, Chaudhry, Oren |  |  |  |  |  |  | X |  |
| Terrell 2009 | Hemens, McKibbon |  |  |  |  |  | X |  |  |
| Terrell 2010 | Jones |  |  |  |  |  | X |  |  |
| Terrell 2011 | Literature search |  |  |  |  |  |  | X |  |
| Theil 1993 | Durieaux |  |  |  |  |  |  | X |  |
| Thompson 2004 | Weir, Van Rosse |  |  |  |  |  | X |  |  |
| Thompson 2005 | Eslami |  |  |  |  |  |  |  | Reporting |
| Thompson 2007 | Hemens |  |  | X |  |  |  |  |  |
| Tierney 1993 | Eslami, Weir, Mollon, Shekelle, Oren, McKibbon, Kawamoto |  |  |  |  |  | X |  |  |
| Tierney 1986 | Chaudhry |  |  | X |  |  |  |  |  |
| Tierney 2003 | Shojania 2009, Shojania 2010, Pearson, Hemens |  |  | X |  |  |  |  |  |
| Tierney 2005 | Goldzweig, Shojania 2009, Shojania 2010, Pearson, Hemens, Nies |  |  | X |  |  |  |  |  |
| Traugott 2011 | Literature search, Jones |  |  |  |  |  |  | X |  |
| Upperman 2005 Pediatrics | Literature Search – hand search |  |  |  | X |  |  |  |  |
| Upperman 2005 J. Pediatric Surg | Shamliyan, van Rosse, Weir, Eslami, Wolfstadt |  |  |  | X |  |  |  |  |
| Vadher 1997 | Pearson, Durieaux , Garg |  |  |  |  |  |  | X |  |
| Van der Sijs 2006 | Literature Search – hand search |  |  |  |  | X |  |  |  |
| Vaidya 2006 | Reckmann |  |  |  | X |  |  |  |  |
| ***Van Doormal 2009*** | Literature search, Manias | Included |  |  |  |  |  |  |  |
| Van Wijk 2001 | Kawamoto |  |  |  |  |  | X |  |  |
| Van Wyk 2008 | Shojania 2009, Shojania 2010, Hemens, McKibbon |  |  | X |  |  | X |  |  |
| Verner 1992 | Pearson, Durieaux |  |  |  |  |  |  | X |  |
| Verstappen 2007 | Hemens |  |  | X |  |  |  |  |  |
| Voeffray 2006 | Reckmann |  |  |  |  |  |  | X |  |
| Walsh 2008 | Hemens, Radley |  |  |  | X |  |  |  |  |
| Wang 2012 | Jones |  |  |  |  |  |  | X |  |
| Warrick 2011 | Literature search |  |  |  | X |  |  |  |  |
| Warshawsky 1994 | Weir |  |  | X |  |  |  |  |  |
| ***Weant 2007*** | Literature search, Manias, Van Rosse | Omitted, no variance info |  |  |  |  |  |  |  |
| Weiner 1999 | Eslami |  |  |  |  | X |  |  |  |
| Weir 2003 | Hemens, McKibbon |  |  | X |  |  |  |  |  |
| Welch 2007 | Weir |  |  | X |  |  |  |  |  |
| Wells 2003 | Shekelle |  |  |  |  |  |  | X |  |
| Wentzer 2007 | Literature search |  |  |  |  | X |  |  |  |
| ***Wess 2007*** | Weir, Literature search | Included |  |  |  |  |  |  |  |
| Westbrook 2009 | Buntin |  |  |  |  |  | X |  |  |
| ***Westbrook 2012*** | Literature search, Jones | Included |  |  |  |  |  |  |  |
| Westbrook 2009 | Literature search |  |  |  |  |  | X |  |  |
| Wetterneck 2011 | Literature search, Jones |  |  |  |  |  |  | X |  |
| White 1984 | Chaudhry, McKibbon |  |  |  |  |  |  | X |  |
| White 1987 | Durieaux, Garg |  |  |  |  |  |  | X |  |
| White 1991 | Durieaux, Garg |  |  |  |  |  |  | X |  |
| Wietholter 2009 | Literature search |  |  |  |  |  | X |  |  |
| Wright 2009 | Literature search |  |  |  |  | X |  |  |  |
| Wright 2012 | Literature search |  |  |  |  | X |  |  |  |
| Wolfenden 2005 | Hemens |  |  | X |  |  |  |  |  |
| Wong 2003 | Chaudhry |  |  |  |  |  | X |  |  |
| Wu 2007 | Literature search |  |  |  |  |  | X |  |  |
| Yu 2009 | Buntin |  |  |  |  | X |  |  |  |
| Zanetti 2003 | Shojania 2009, Shojania 2010, Hemens, McKibbon |  |  |  |  |  |  | X |  |
| Zlabek 2011 | Literature search, Jones |  |  |  |  |  |  |  | Reporting |

* Includes events not meeting study definitions of MEs or ADEs.

**Appendix Table 3: Additional Characteristics of Included Studies**

| **Study, Year (Citation)** | **Study Design** | **Event Detection Methods** | **Quality of Event Detection by Reviewers** | **Funding Source** |
| --- | --- | --- | --- | --- |
|
| **No CDSS** | | | | |
| Bizovi et al. 2002 | Pre/Post | Routine pharmacist review of medication orders | Training: Not stated  Blinding: Not stated  Reliability: Not stated | U.S. federal grant |
| Franklin et al. 2009 | Pre/Post | Routine pharmacist review of medication orders, medical record review and incident reporting | Training: Not stated  Blinding: Not stated  Reliability: Not stated | UK governmental grant |
| Shawahna et al. 2011 | Pre/Post | Medical record review | Training: Not stated  Blinding: Not stated  Reliability: Assessed | Not stated |
| Shulman et al. 2005 | Pre/Post | Routine pharmacist review of medication orders | Training: Not stated  Blinding: Not stated  Reliability: Not stated | Not stated |
| **CDSS Present** | | | | |
| Leung et al. 2012 | Pre/Post | Medical record and order review | Training: Described  Blinding: Yes  Reliability: Assessed | Private foundations |
| Wess et al. 2007 | Pre/Post | Routine pharmacist review of medication orders, with changes signed by MD | Training: Not stated  Blinding: Not stated  Reliability: Not stated | Private foundation |
| Taylor et al. 2002 | Pre/Post | Quarterly review of subset of medication orders | Training: Not stated  Blinding: Not stated  Reliability: Not stated | Not stated |
| Weant et al. 2007 | Pre/Post | Routine pharmacist review of medication orders, incident reporting | Training: Not stated  Blinding: Not stated  Reliability: Not stated | Not stated |
| Barron et al. 2006 | Pre/Post | Routine pharmacist review of medication orders | Training: Not stated  Blinding: Not stated  Reliability: Not stated | Study institution |
| Bates et al. 1998 | Pre/Post | Medical record review and other means | Training: Not stated  Blinding: Yes  Reliability: Assessed | Private foundations, Veterans Affairs |
| Van Doormal et al. 2009 | Pre/Post | Medical record and order review | Training: Not stated  Blinding: Not stated  Reliability: Not stated | Dutch governmental grant |
| Westbrook et al. 2012 | Differences-in-  differences | Routine pharmacist review of medication orders | Training: Not stated  Blinding: Not stated  Reliability: Assessed | Australian governmental grant |
| Bates et al. 1999 | Pre/Post | Medical record and order review plus other means | Training: Not stated  Blinding: Not stated  Reliability: Assessed | Private foundation |
| Colpaert et al. 2006 | Compare similar units | Routine pharmacist review of medication orders | Training: Not stated  Blinding: Yes  Reliability: Not stated | Not stated |
| Mahoney et al. 2007 | Pre/Post | Routine pharmacist review of medication orders, with changes accepted by MD; incident reporting | Training: Not stated  Blinding: Not stated  Reliability: Not stated | Not stated |
| Oliven et al. 2005 | Compare similar units | Medical record and order review | Training: Not stated  Blinding: Not stated  Reliability: Not stated | Not stated |
| Igboechi et al. 2003 | Pre/Post | Routine pharmacist review of medication orders | Training: Not stated  Blinding: Not stated  Reliability: Not stated | Not stated |
| Aronsky et al. 2007 | Pre/Post | Routine pharmacist review of medication orders | Training: Not stated  Blinding: Not stated  Reliability: Not stated | U.S. federal grant |
| Mendendez et al. 2012 | Pre/Post | Trigger tool medical record review, incident reporting, and other means | Training: Not stated  Blinding: Not stated  Reliability: Not stated | Not stated |

**Appendix Table 4: Methods Used to Identify Medication Errors (MEs) and pADEs in Each Study, Paper Order Entry vs. CPOE**

| **Definition of ME / Event Detection Methods from Study** | **Paper-Order Entry, MEs or pADEs per Unit of Exposure** | **CPOE, MEs or**  **pADEs per Unit of Exposure** | **Study** |
| --- | --- | --- | --- |
| Nurse investigator and physician reviewer detected MEs; study only reported non-intercepted serious MEs (argued that intercepted MEs cannot be prevented by CPOE, but this makes study less comparable to others, which generally used events intercepted by pharmacist as outcome measure) | 127 non-intercepted serious ME / 12,218 patient-days  55 pADEs / 12,218 patient-days | 54 non-intercepted serious ME / 11,235 patient-days  41 pADEs / 11,235 patient-days | Bates 1998 |
| Nurse investigator and physician reviewer detected MEs excluding missed doses of medications. Study reported both intercepted and non-intercepted MEs; we did not exclude intercepted MEs. | 242 non-missed dose MEs /  1704 patient-days  5 pADEs /  1704 patient-days | Period 3, after implementation has stabilized:  50 non-missed dose MEs / 1878 patient days  2 pADEs / 1878 patient-days | Bates 1999 |
| Pharmacist detected MEs by reviewing orders. MEs excluded “minor,” defined as those with no potential for harm  ADEs had potential to cause or actually caused harm, definition did not mention being intercepted | (331 prescribing errors – 225 “minor” errors) =  106 ME /  1,224 orders or 80 patient-days  12 ADEs /  1,224 orders or 80 patient-days | (44 prescribing errors – 9 “minor” errors) =  35 ME /  1,286 orders or 80 patient-days  2 ADEs /  1,286 orders or 80 patient-days | Colpaert 2006 |
| Pharmacist detected prescribing and transcribing errors; study did not include pADEs | (183 illegible +  750 incomplete +  1884 incorrect +  1127 problems) =  3,944 ME / 14,907 hospitalizations in Period 2 | (38 illegible +  200 incomplete +  1009 incorrect +  1081 problems) =  2,328 ME / 14,352 hospitalizations in Period 3 | Igboechi 2003 |
| Nurse investigator and physician reviewer detected MEs; potential ADEs (medication errors that did not cause harm) + preventable ADEs including intercepted potential ADEs | (106 preventable ADEs +444 potential ADEs) =  550 ME/ 1000 hospitalizations  106 preventable ADEs  / 1000 hospitalizations | (70 preventable ADEs + 575 potential ADEs) =  645 ME/ 1000 hospitalizations  70 preventable ADEs  / 1000 hospitalizations | Leung 2012 |
| Pharmacist “interventions” that were accepted by physicians leading to an order change, incident reporting, review of electronic administration record error reports | (833 drug allergy +  1341 excessive dose +  665 therapeutic duplic +  1976 incompl/unclear ord +  44 colchicine dose +  101 metformin dose)=  4960 ME /  1,452,346 orders | (109 drug allergy+  871 excessive dose+  584 therapeutic duplic +  663 incompl/unclr ord +  26 colchicine dose +  66 metformin dose) =  2319 ME /  1,390,789 orders | Mahoney 2007 |
| Resident-physician detected prescribing errors | ([0.41 Type 1 + 0.56 Type 2]  = 0.97 ME / patient)*641 patients  /4969 hospitalization days | ([0.10 Type 1 + 0.21 Type 2] = 0.31 ME / patient)*709 patients  /5033 hospitalization days | Oliven 2005 |
| Pharmacist detected prescribing and transcribing errors;  Allows more than one error per order. Unclear whether one error can go into more than one causation category. | 3,971 Medication orders with ME /  592 admissions or (3315+3971 medication orders)  92 patients with pADEs or (34 + 55 + 15 = 104 errors related to pADEs)/  592 admissions or (3315+3971 medication orders) | 1,207 medication orders with ME /  603 admissions or (5855+1203 medication orders)  44 patients with pADEs or (29 + 23 + 1 = 53 errors related to pADEs)/  603 admissions or (5855+1203 medication orders) | Van Doormal 2009 |
| Pharmacist detected prescribing errors:  Pre-Post Data from Hospitals A & B (ignoring control data from Hospital A). | 4270 prescribing errors = (1,094 Intervention 1 +  337 Intervention 2 +  2,839 Intervention 3) /  1053 admissions =  (175 Intervention 1 +  77 Intervention 2 +  801 Intervention 3) | 1029 prescribing errors = (348 Intervention 1 +  119 Intervention 2 +  562 Intervention 3) =  / 629 admissions =  (164 Intervention 1 +  64 Intervention 2 +  401 Intervention 3) | Westbrook 2012 |
| Emergency Dept: Potential ADEs and medication prescribing errors involving illegible or missing info; we ignored rule violations, which were JCAHO issues about leading/trailing zeros; study did not include pads.  One error per order. | 125 potential ADEs / 3383 orders | 73 potential ADEs / 2567 orders | Aronsky 2007 |
| Pharmacist detected prescribing and transcribing errors; study did not include pADEs | 0.71 Prescribing plus 0.34 Transcribing errors = 1.05 per 1,000 doses  doses dispensed per 240,096 month  Medication Errors =(1.05/1000)*240,096=252 | 0.31 Prescribing plus 0.01 Transcribing errors = 0.32 per 1,000 doses  240,096 doses dispensed per month  Medication Errors =(0.32/1000)*240,096=77 | Barron 2006 |
| Emergency Dept: Pharmacist detected ordering errors and potential ADEs | 54 ME / 2,326 prescriptions for 1,459 patients | 11 ME / 1,594 prescriptions for 1,056 patients | Bizovi 2002 |
| Pharmacist detected prescribing errors, chart review, spontaneous reporting, trigger tool | 135 prescribing errors /  438 patient days | 127 prescribing errors /  501 patient days | Franklin 2009 |
| Incident reporting, Global Trigger Tool (presumably chart review), and pharmacist walk rounds  pADEs: Called NCCMERP categories E to I “moderate to severe errors” | 356 errors/  7,001 discharges  33 moderate to severe errors (E-I)/  7,001 discharges | 1197 errors/  11,347 discharges  11 moderate to severe errors (E-I)/  11,347 discharges | Mendendez  2012 |
| Prescribing and transcribing errors judged by panel | 3,008 ME / 13,328 orders | 1,147 ME / 14,064 orders | Shawahna 2011 |
| Pharmacist detected prescribing and transcribing errors | 71 ME / 1,036 orders | 117 ME / 2,429 orders | Shulman 2005 |
| Pharmacy team and medical staff reviewed 2,500 to 3,000 medication orders per quarter | N/A | 50% decline in ME | Taylor 2002 |
| Study did not use term medication error. Studied routine pharmacist review of medication orders, with changes signed by MD “Calls to MD requiring signature” | (133 calls at community hospital + 106 calls at university hospital) =  239 ME / 8,595 orders  (4755 orders at community hospital + 3840 at university hospital) | (23 calls at community hospital + 34 calls at university hospital) =  57 ME / 13,105 orders  (5775 orders at community hospital + 7330 at university hospital) | Wess 2007 |
| “Pharmacist interventions” and voluntary incident reporting data | 0.938 No-Harm Errors (Categories B-D) +  0.137 Harmful Errors (Categories E-H) =  1.075 ME / 1000 doses  0.137 Harmful Errors (Categories E-H) / 1000 doses | 1.839 No-Harm Errors (Categories B-D) +  0.0152 Harmful Errors (Categories E-H) =  1.8542 ME / 1000 doses  0.0152 Harmful Errors (Categories E-H) / 1000 doses | Weant 2007 |

**Appendix Table 5: Implementation Information from Studies in Pooled Analysis**

| **Question** | Aron-sky 2007 | Barron 2006 | Bates 1998 | Bates 1999 | Bizovi 2002 | Col-paert 2006 | Frank-lin 2009 | Leung  2012 | Maho-ney 2007 | Men-dendez 2012 | Oliven 2005 | Sha-wahna 2011 | Shul-man 2005 | Van Door-mal 2009 | Wess 2007 | West-brook 2012 |
| --- | --- | --- | --- | --- | --- | --- | --- | --- | --- | --- | --- | --- | --- | --- | --- | --- |
| **Organizational Characteristics** |  |  |  |  |  |  |  |  |  |  |  |  |  |  |  |  |
| Complexity* | ? | ? | Com-plex | Com-plex | Large | ? | . | Com-munity | Com-plex | . | . | ? | ? | . | Com-plex | . |
| Physician Ownership† | No | No | No | No | No | No | No | . | No | No | No | No | No | No | . | No |
| Existing Quality/Safety Infrastructure | . | Yes | . | . | . | . | Yes | . | Yes | . | . | ? | . | . | Yes | . |
| Past Experience with IT | . | Other IT | Other IT | Other IT | Other IT | ? | Replac-ing EHR | . | Other IT | . | . | . | . | . | Other IT | . |
| **Influenced Decision** |  |  |  |  |  |  |  |  |  |  |  |  |  |  |  |  |
| Regulatory Requirements | . | . | . | . | . | . | . | . | . | . | . | . | . | . | . | . |
| Externally Imposed Payments or Penalties | . | . | . | . | . | . | . | . | . | . | . | . | . | . | . | . |
| Organizational Leadership | . | Yes | . | . | . | . | . | . | Yes | . | . | . | . | . | Yes | . |
| **Facilitated Implementation** |  |  |  |  |  |  |  |  |  |  |  |  |  |  |  |  |
| Staff Education and Training | . | Yes | . | . | . | . | . | . | . | . | Yes | Yes | Yes | . | . | . |
| Designated Staff Time to Learn CPOE | . | Yes | . | . | . | . | . | . | . | . | . | . | . | . | . | . |
| Internal or External Responsible Person | . | Yes | . | . | . | . | . | . | Yes | . | . | . | . | . | . | . |
| Internal  Incentives | . | . | . | . | . | . | . | . | . | . | . | . | . | . | . | . |
| Local Tailoring or an Iterative Process | . | . | . | . | . | . | . | . | Yes | . | . | . | . | . | . | . |
| Helpdesk  Support | . | . | . | . | . | . | . | . | . | . | . | . | . | . | . | . |
| Extensive Project Management | . | Yes | . | . | . | . | . | . | . | . | . | . | . | . | . | . |
| Implementation Timeline | . | Yes | . | . | . | . | . | . | . | . | . | . | . | . | . | . |
| At-once or Rolling Implementation | (One unit) | At-once | . | Rolling | (One unit) | At-once | (One unit) | . | . | . | (One unit) | At-once | (One unit) | . | Rolling | At-once |
| **Aspects Described** |  |  |  |  |  |  |  |  |  |  |  |  |  |  |  |  |
| Implementation Process | . | . | . | . | . | . | . | . | . | . | . | Yes | . | . | . | . |
| Unexpected Aspects of Implementation | . | . | . | . | . | . | . | . | . | . | . | . | . | . | . | . |
| Teamwork in the Organization re: CPOE | . | Yes | . | . | . | . | . | . | Yes | . | . | . | . | . | . | . |
| Patient Safety Culture in Organization re: CPOE | . | Yes | . | . | . | . | . | . | . | . | . | . | . | . | . | . |

* “?” = Unable to classify based on available information, “.” = No information available.

† Assumed that academic hospitals were not physician owned.

**Appendix Figure 1: Funnel Plot of Six Studies Included in Meta-Analysis of CPOE’s Effect on pADEs**

**
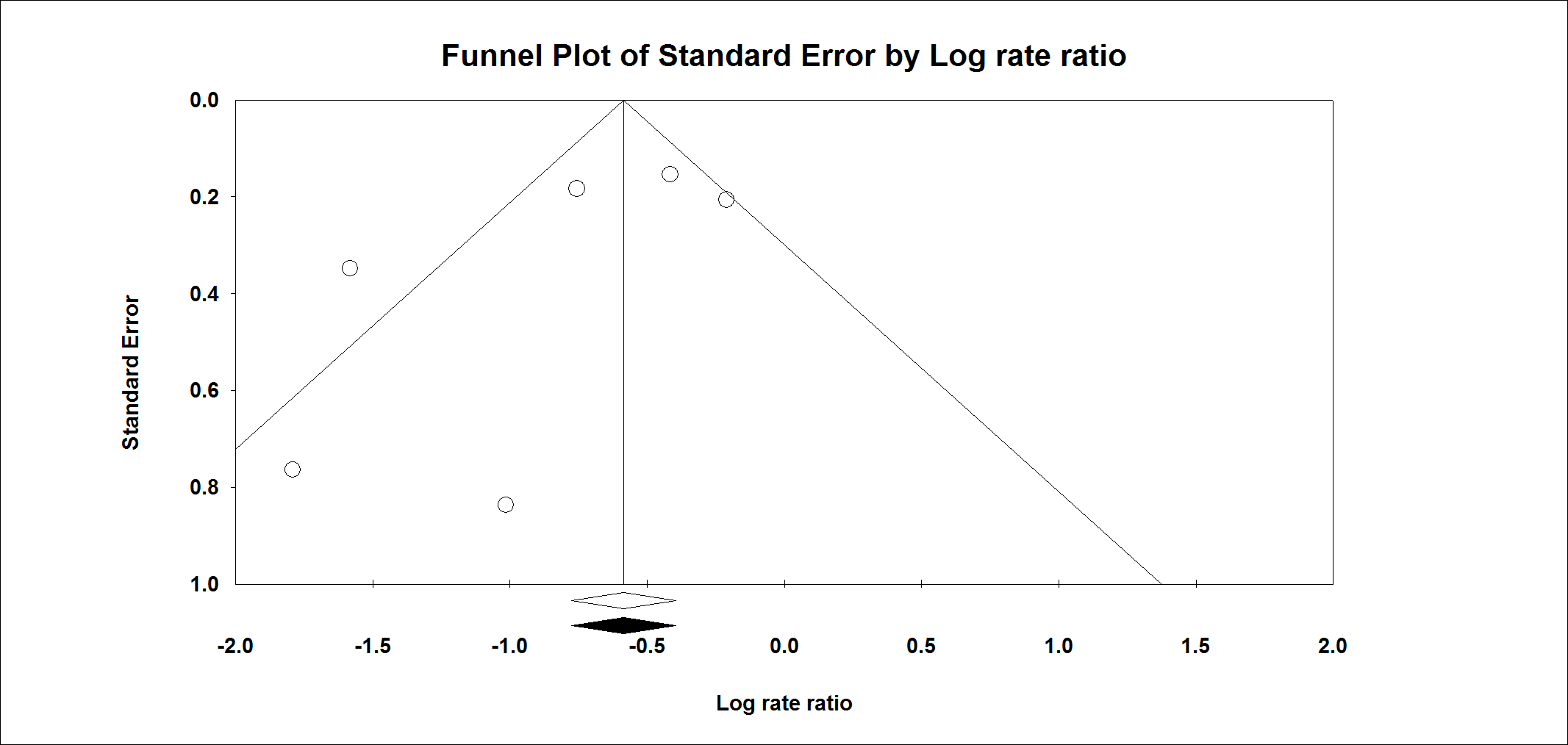
**

**Begg and Mazumdar’s test (Kendall’s tau = -0.13, z = 0.38, p = 0.71)**

**Egger’s test (95%-CI -5.85 to 1.61, p = 0.19)**

**Appendix Figure 2: Funnel Plot of 16 Studies Included in Meta-Analysis of CPOE’s Effect on Medication Errors**

**
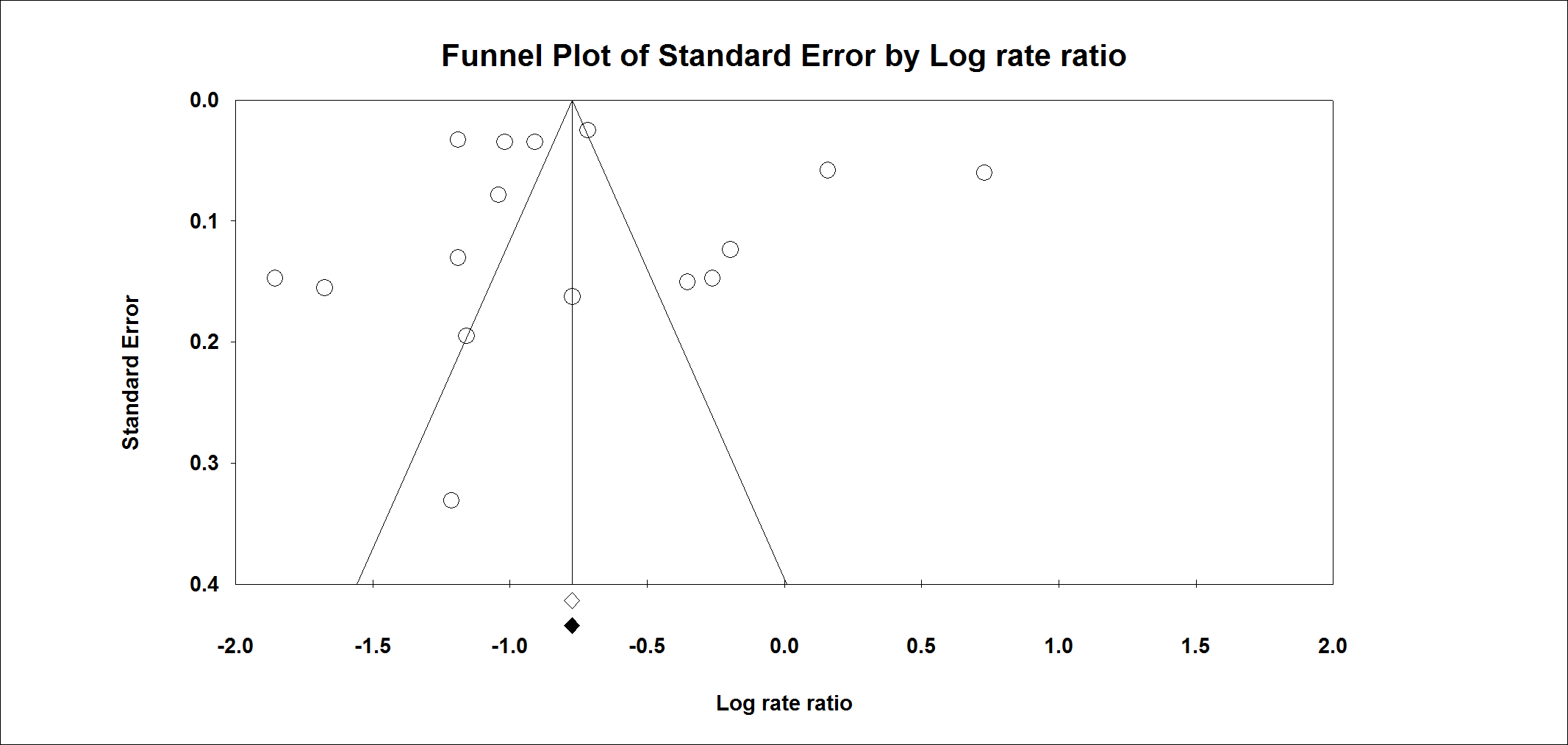
**

**Begg and Mazumdar’s test (Kendall’s tau with continuity correction=0.06, z = 0.32, p = 0.75)**

**Egger’s test (95%-CI -7.02 to 9.71, p = 0.74)**

**Appendix Table 4: Comparison of Studies Included in Previous Meta-Analyses and Current Analysis, vs. Included in Previous Meta-Analyses or Systematic Reviews and Excluded from Current Analysis**

| **Authors of Previous Analysis** | **Studies in Previous Analysis, Number** | **Studies Included in Both Previous Analysis and Current Study,**  **First Author and Year** | **Studies Excluded from Current Study But Included in Previous Analysis, Number and Reason** |
| --- | --- | --- | --- |
| Radley et al. (2013) | 8 | Bizovi 2002  Bates 1999  Mahoney 2007 | **Excluded from Study:**  1 unique type of events  4 pediatric settings |
| Van Rosse et al. (2009) | 3 | **Medication Errors:**  Colpaert 2006  Shulman 2005 | **Excluded from Study:**  1 pediatric setting  (ADEs—All 3 studies excluded due to pediatric settings) |
| Shamliyan et al. (2008) | 6 | Bates 1999  Bates 1998  Shulman 2005  Oliven 2005  Bizovi 2002 | **Excluded from Study:**  1 outpatient setting |
| Ammenwerth et al. (2008)  (did not do a pooled meta-analysis) | 25 | **Medication Errors:**  Colpaert 2006  Bates 1999  Shulman 2005  Bizovi 2002  Oliven 2005  **pADEs:**  Colpaert 2006  Bates 1999  Bates 1998 | **Excluded from Study:**  3 pediatric settings  12 unique types of events  2 outpatient settings,  1 event detection methods  **Excluded from Pooled Analysis:**  Igboechi 2003-variance data  Mitchell 2004-variance data |

1. Ogrinc G, Mooney SE, Estrada C, Foster T, Goldmann D, Hall LW, Huizinga MM, Liu SK, Mills P, Neily J, Nelson W, Pronovost PJ, Provost L, Rubenstein LV, Speroff T, Splaine M, Thomson R, Tomolo AM, Watts B. The SQUIRE (Standards for QUality Improvement Reporting Excellence) guidelines for quality improvement reporting: explanation and elaboration.Qual Saf Health Care. 2008 Oct;17 Suppl 1:i13-32. doi: 10.1136/qshc.2008.029058. [↑](#footnote-ref-2)
2. Paul G. Shekelle, M.D., Ph.D.; Peter J. Pronovost, M.D., Ph.D.; Robert M. Wachter, M.D. Assessing the Evidence for Context-Sensitive Effectiveness and Safety of Patient Safety Practices: Developing Criteria. Prepared for: Agency for Healthcare Research and Quality, U.S. Department of Health and Human Services, 540 Gaither Road, Rockville, MD 20850. [www.ahrq.gov](http://www.ahrq.gov/). Contract No. HHSA-290-2009-10001C. Available at: <http://www.ahrq.gov/qual/contextsensitive/> [↑](#footnote-ref-3)
